# A Versatile Intestine‐on‐Chip System for Deciphering the Immunopathogenesis of Inflammatory Bowel Disease

**DOI:** 10.1002/adhm.202302454

**Published:** 2024-02-11

**Authors:** Oanh T. P. Nguyen, Patrick M. Misun, Andreas Hierlemann, Christian Lohasz

**Affiliations:** ^1^ Bio Engineering Laboratory Department of Biosystems Science and Engineering ETH Zurich Klingelbergstrasse 48 Basel CH‐4056 Switzerland

**Keywords:** immunopathogenesis, inflammatory bowel disease, innate immunity, intestine‐on‐chip systems

## Abstract

The multifactorial nature of inflammatory bowel disease (IBD) necessitates reliable and practical experimental models to elucidate its etiology and pathogenesis. To model the intestinal microenvironment at the onset of IBD in vitro, it is important to incorporate relevant cellular and noncellular components before inducing stepwise pathogenic developments. A novel intestine‐on‐chip system for investigating multiple aspects of IBD's immunopathogenesis is presented. The system includes an array of tight and polarized barrier models formed from intestinal epithelial cells on an in‐vivo‐like subepithelial matrix within one week. The dynamic remodeling of the subepithelial matrix by cells or their secretome demonstrates the physiological relevance of the on‐chip barrier models. The system design enables introduction of various immune cell types and inflammatory stimuli at specific locations in the same barrier model, which facilitates investigations of the distinct roles of each cell type in intestinal inflammation development. It is showed that inflammatory behavior manifests in an upregulated expression of inflammatory markers and cytokines (TNF‐α). The neutralizing effect of the anti‐inflammatory antibody Infliximab on levels of TNF‐α and its inducible cytokines could be explicitly shown. Overall, an innovative approach to systematically developing a microphysiological system to comprehend immune‐system‐mediated disorders of IBD and to identify new therapeutic strategies is presented.

## Graphical abstract

A novel intestine‐on‐chip microfluidic system for parallel and reproducible formation of multiple physiologically relevant in vitro intestinal barrier models is presented. The system enables simultaneous multiparameter investigations of the role of different immune cell types in intestinal inflammation and of their specific responses to anti‐inflammatory drug therapy through high‐resolution microscopy, analysis of cell surface markers, and chemo/cytokine profiling.

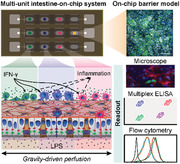

## 1 Introduction

Inflammatory bowel disease (IBD) is an umbrella term for two distinct disorders – Crohn's disease (CD) and ulcerative colitis (UC) – that feature chronic and relapsing inflammation of the gastrointestinal tract.^[^ ^1^, ^2^, ^3^ ^]^ In 2017, 6.8 million cases of IBD were recorded globally – mostly in high‐income countries in North America and Europe.^[^ ^4^ ^]^ IBD patients suffer from recurring flare‐ups that include abdominal pain, fatigue, weight loss, diarrhea accompanied by blood, or, in rare cases, life‐threatening complications.^[^ ^5^ ^]^ The etiology of IBD is multifactorial, and much of it remains unclear. Due to this knowledge gap, existing IBD treatments mainly address the symptoms to support partial remission. The first‐line IBD therapy – the tumor necrotic factor (TNF) blockers – fail to induce therapeutic responses in up to 40% of the patients, and ≈23–46% of the patients experience the loss of therapeutic responses after one year.^[^ ^6^, ^7^ ^]^ Although most IBD treatments are generally well tolerated, unforeseen and serious adverse events still occur in clinical treatments, leading to therapy discontinuation in more than 20% of the patients.^[^ ^8^ ^]^

IBD is an immune‐mediated disease and has been shown to involve inappropriate immune responses against the intestinal microflora in genetically susceptible individuals. In homeostasis, the intestinal immune system balances immune tolerance and inflammation in response to changes in the intestinal microflora.^[^ ^9^ ^]^ This process is primarily governed by intestinal mononuclear phagocytes (MNPs), comprising monocytes, macrophages, and immature dendritic cells,^[^ ^9^, ^10^, ^11^ ^]^ and effector T helper (i.e., CD4^+^) cells.^[^ ^12^ ^]^ Recent research has linked defects in MNP‐driven immune responses to intestinal inflammation and IBD susceptibility,^[^ ^9^, ^10^, ^11^, ^13^, ^14^ ^]^ while CD4^+^ T cells have been suggested as the major disease initiators.^[^ ^15^ ^]^ More insights into specific behaviors of each immune cell population during the onset of IBD will greatly benefit the development of new symptom‐management approaches and therapeutic treatments.

Throughout the years, different in vivo, ex vivo, and in vitro models have been developed to investigate the pathogenesis of IBD and to test new treatment strategies. In vivo animal models, such as genetically modified or inflammatory inducer‐treated mice, rats, guinea pigs, rabbits, pigs, dogs, and nonhuman primates,^[^ ^16^ ^]^ support systemic investigations of the involvement of the intestinal microflora and immune system in IBD pathogenesis. However, systemic complexity often limits mechanistic studies of disease‐relevant pathways. Furthermore, the use of animal models entails ethical aspects, low throughput, and, most importantly, experimental outcomes require cautious interpretation due to critical inter‐species differences in the immune system and the intestinal microbiome.^[^ ^17^, ^18^ ^]^ Ex vivo human intestinal explants, in particular IBD patient‐derived samples, closely recapitulate the in vivo intestinal structure and can provide valuable insights into patient heterogeneity in terms of involved immune components and drug responses. The largest limitations of such models remain their scarcity and limited lifespan in ex vivo cultures (maximum 24 hours under optimal conditions).^[^ ^19^ ^]^ In the majority of academic laboratories, intestinal organoids and transwell‐based cell models are commonly used for IBD research, because these models are easy to access and handle using standard laboratory methods. Intestinal organoids offer in vivo‐like architectures and reproduce the cellular complexity of the intestinal epithelial barrier (IEB). However, the apical side of the barrier is enclosed inside the organoid body and has limited accessibility to manipulation or analysis. Static transwell‐based cell models require long maturation times to form polarized IEB models (21 days). Moreover, these IEB models do not undergo self‐organization and fail to reproduce the 3D morphology that is characteristic of the in vivo IEB.

Novel strategies to investigate the pathogenesis of IBD and treatment responses under more physiologically relevant conditions have recently been introduced in the form of human cell‐based intestine‐on‐chip systems.^[^ ^20^, ^21^ ^]^ In these systems, an IEB model is typically developed by growing one or two of the most abundant cell types of the in vivo IEB model (i.e., enterocytes and mucus‐producing goblet cells) on a supporting substrate, such as a porous, extracellular matrix (ECM)‐coated artificial membrane^[^ ^22^ ^]^ or an ECM hydrogel column.^[^ ^23^ ^]^ The artificial membrane or hydrogel separates the apical side (lumen) of the IEB model from its basal side (subepithelial stromal niche), where other cell types, such as fibroblasts, immune cells, and endothelial cells reside. In such systems, key mechanical cues – most importantly shear stress through fluidic flow – can be induced to promote the development of the IEB models by providing i) a constant medium turnover and ii) shear stress that the epithelial cells of the IEB need for proper differentiation.^[^ ^24^ ^]^ Systems with a fluidic flow have proven to shorten the time needed to develop a polarized barrier model from 21 days to less than 7 days.^[^ ^25^ ^]^ Depending on the scientific question of interest, different stimuli can be added to either side of the IEB model to trigger the development of certain phenotypes. Common stimuli used for modeling IBD in intestine‐on‐chip systems are i) dextran sodium sulfate (DSS) and/or microbial components (e.g., lipopolysaccharide (LPS) endotoxin, live bacteria) on the apical side, and/or ii) pro‐inflammatory cytokines on the basal side.^[^ ^21^, ^26^, ^27^, ^28^ ^]^ As a prime example, an “intestine inflammation‐on‐a‐chip” system was developed to study interactions between an on‐chip IEB model, peripheral blood mononuclear cells (PBMCs), and microbial components (nonpathogenic bacteria or LPS) during the onset of intestinal inflammation.^[^ ^28^ ^]^ The authors reported LPS‐ and PBMC‐dependent pathogenic inflammation only when the IEB model's integrity was compromised with DSS.^[^ ^28^ ^]^ As it is unclear whether a compromised intestinal barrier is the cause or consequence of intestinal inflammation,^[^ ^29^ ^]^ novel intestine‐on‐chip systems that can support investigations on immunopathogenesis and inflammation‐induced changes of the IEB are needed.

Although existing intestine‐on‐chip systems proved their relevance for modeling IBD in vitro, they still feature several limitations. Polydimethylsiloxane (PDMS) – the most commonly used material to prototype microfluidic chips – ad/absorbs a wide range of molecules and features the problem of evaporation,^[^ ^30^, ^31^ ^]^ which renders the use of PDMS‐based systems for drug‐testing experiments challenging. Artificial porous membranes, which can be fabricated from PDMS, polyester, or polycarbonate, feature high stiffness, in contrast to the elastic in vivo basement membranes.^[^ ^32^ ^]^ Cells grown on these substrates have been shown to exhibit altered migration, proliferation, and differentiation.^[^ ^33^ ^]^ Other intestine‐on‐chip systems that were based on non‐PDMS materials, such as glass or thermoplastics, and/or included in vivo‐like basement membranes helped to resolve these PDMS‐associated problems.^[^ ^34^, ^35^ ^]^ However, such systems require long prototyping times and offer limited design flexibility. Therefore, novel non‐PDMS systems that i) feature the formation of IEB models on in vivo‐like ECM substrates, ii) have short prototyping times, and iii) are scalable in their operation are urgently required.

**Figure 1:**
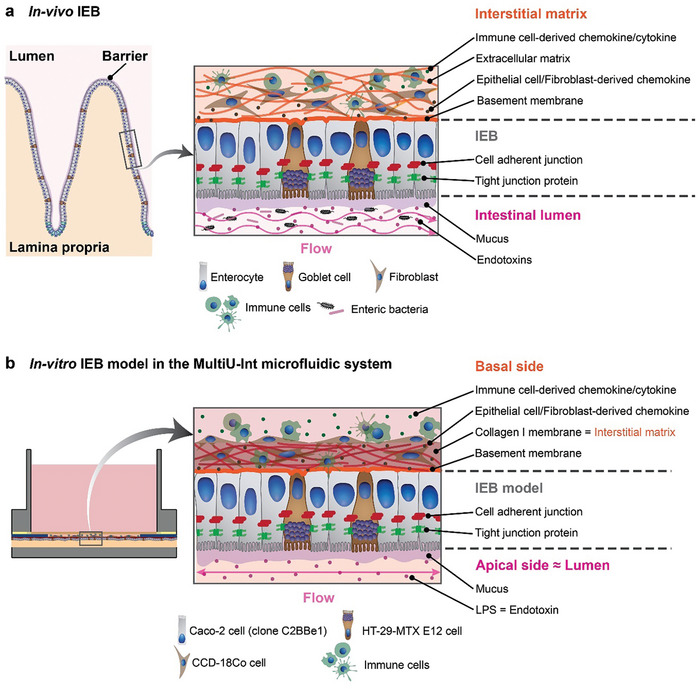
Schematic representation of key structural and cellular components of a) the in vivo IEB and b) an in vitro IEB model formed within the MultiU‐Int microfluidic chip.

In this work, we present a novel, multiunit intestine‐on‐chip system – MultiU‐Int – that enables investigations of the involvement of different immune cell populations and inflammatory stimuli in the initiation of IBD. The body of our microfluidic chip was fabricated from thermoplastics to avoid compound absorption and medium evaporation. An elastic collagen I membrane – formed from native, non‐crosslinked collagen fibers – was used as a physiologically relevant scaffold to support the intestinal–epithelial–cell (IEC) layer. This bioresponsive membrane allows for robust cell–cell and cell–matrix interactions and supports cellular growth and differentiation.^[^ ^36^, ^37^, ^38^ ^]^ Each on‐chip IEB model features multiple spatially separated basal compartments, in which different immune cell types or cytokine stimuli can be independently added or applied. Shear stress was realized through liquid flow by gravity‐driven perfusion, making chip usage and operation scalable and user‐friendly. Key cellular and architectural components of intestines in a human body and their implementation in the MultiU‐Int microfluidic chip are schematically represented in **Figure** **1**. Using this system, we performed parallelized screening for inflammation‐initiating immune cells by i) individually loading distinct immune cell populations into the basal compartments, ii) applying a clinically relevant level of LPS on the apical side of the IEB model,^[^ ^39^ ^]^ and iii) selectively adding interferon‐γ (IFN‐γ) to selected basal compartments to trigger inflammatory conditions.^[^ ^40^, ^41^ ^]^ We then demonstrated the potential of our system for drug testing by treating inflamed IEB model units with Infliximab – one of the most widely used TNF blockers for treating IBD.

## 2 Results

### 2.1 The MultiU‐Int Microfluidic Chip

#### 2.1.1 Design Concept

**Figure 2:**
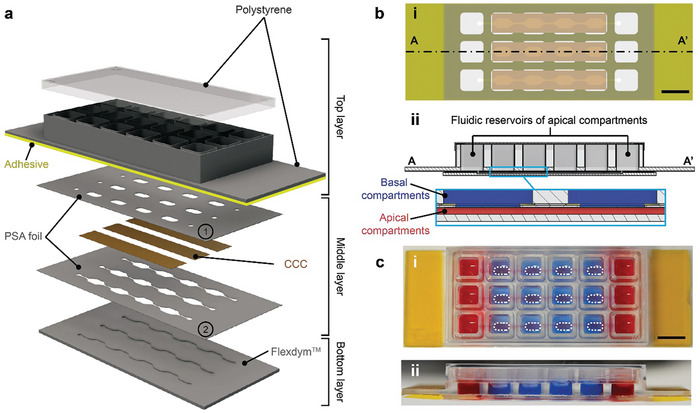
a) Schematic representation of the individual layers of the MultiU‐Int microfluidic chip, their arrangement, and materials. The top layer was a commercially available, multi‐well slide made from polystyrene plastic material (ibidi sticky‐slide 18 well). The middle layer of the chip was fabricated by sandwiching CCC strips between two pressure‐sensitive adhesive (PSA) foils. The upper foil (①) featured oval areas where fibroblasts and, later, immune cells were allowed to interact with the IEB model (fibroblast seeding areas). The lower foil (②) featured the same pattern of channel structures that were hot‐embossed into the bottom layer of the chip. The bottom layer was fabricated by hot embossing the elastomer Flexdym. b) Schematic cross‐sections of the MultiU‐Int microfluidic chip showing details of the layer alignment: i) bottom view, and ii) side view. c‐i) Top view, and c‐ii) side view photographs of the MultiU‐Int microfluidic chip. For visualization, apical channels and their reservoirs were filled with red fluid, and the basal compartments were filled with blue fluid. The cell seeding areas on the basal side are marked with white dashed lines. Scale bars: 10 mm.

The developed MultiU‐Int microfluidic system features i) a realistic IEB model, ii) fluidic and optical access to all compartments, and iii) scalable experimental operation. All the structural parts of the microfluidic chip – top, middle, and bottom layers as shown in **Figure** **2**a – were fabricated from thermoplastic materials. The top layer consisted of polystyrene plastic and featured a grid of 3 × 6 wells (row × column). This grid of wells served two different purposes: i) the two outermost columns on both sides were used as cell culture medium reservoirs that were connected to the apical compartments, and ii) the four middle columns were used as the basal compartments of the on‐chip IEB model. The rigidity of the plastic material rendered the microfluidic chip mechanically stable. The bottom layer was fabricated from a hot‐embossed thermoplastic elastomer, Flexdym,^[^ ^42^ ^]^ and featured microfluidic channels that interconnected the six wells within the same row of the top layer. Flexdym enables fast and highly adaptable prototyping of microfluidic components and its superior characteristics over PDMS, including its low ad/absorption of small hydrophobic molecules have been documented.^[^ ^42^ ^]^ Lastly, the middle layer was sandwiched between the top and bottom layers and featured a collagen I membrane (branded as collagen cell carrier (CCC)), which was formed from native, non‐crosslinked collagen fibers. While the CCC is permeable to most soluble factors in the cell‐culture medium and supernatant, it is impermeable to cells, unless they are highly motile or secreting collagenase. The on‐chip IEB model was formed on the surface of this CCC and separated apical (bottom layer) and basal (top layer) fluidic compartments. The detailed dimensions of the individual parts are shown in Figure S1 (Supporting Information), and a schematic of the bottom‐up fabrication process is shown in Figure S2 (Supporting Information).

As shown in Figure 2b‐i,c‐i, each microfluidic chip featured three individual microfluidic channels in the bottom layer that were arranged in parallel along the long axis of the chip. Each channel constituted one apical compartment of an on‐chip IEB model (Figure 2b‐ii,c‐ii, depicted in red) that had a volume of 22 µL and was accessible through the fluidic reservoirs at both ends. Each apical compartment was connected to four spatially separated basal compartments in the top layer (Figure 2b,c‐ii), depicted in blue) on the other side of the CCC. These four basal compartments could be manipulated individually, meaning that multiple stimuli or immune cell types could be tested simultaneously on the same IEB model. Such a design concept is particularly beneficial for experiments with rare patient‐derived materials. All fluidic reservoirs had a working volume of 100‐210 µL and they all resided in the top layer of the microfluidic chip. Therefore, manipulations, such as cell loading, medium exchange, supernatant sampling, and cell harvesting, were straightforward and could be performed using a multi‐channel pipette.

#### 2.1.2 Operation of the MultiU‐Int Microfluidic Chip

Our MultiU‐Int microfluidic chip had the size of a standard microscopy slide (25 mm width, 75 mm length), so that four chips could be inserted in one standard slide holder or rectangular well plate to conduct experiments in parallel. Perfusion through the apical compartments was induced by gravity‐driven flow. To start the perfusion, we placed the chip holder on a tilting platform and tilted the chip back and forth along its long axis by ± 4°, as shown in Figure S3a (Supporting Information). Based on computational simulations, a maximum flow rate of 134 µL min^−1^ and an average flow‐induced shear stress of 0.025 Pa at the apical surface of the IEB model were achieved at the indicated tilting angle. The calculated shear stress in our system was within the range of physiologically relevant shear stresses between 0.0002 and 0.08 Pa for in vivo IEBs.^[^ ^21^, ^43^ ^]^ Additionally, we included a resting period of 58 min between the tilting cycles to simulate the quiescent period of the motility pattern during the interdigestive period of the small intestine.^[^ ^44^ ^]^ Figure S3b,c (Supporting Information) shows the arrangement of the MultiU‐Int chips on the tilting device and a simulation of the average fluid shear stress at the IEB.

### 2.2 Development of the On‐chip Intestinal Epithelial Barrier Model

#### 2.2.1 Sequential Establishment of the IEB Model

**Figure 3:**
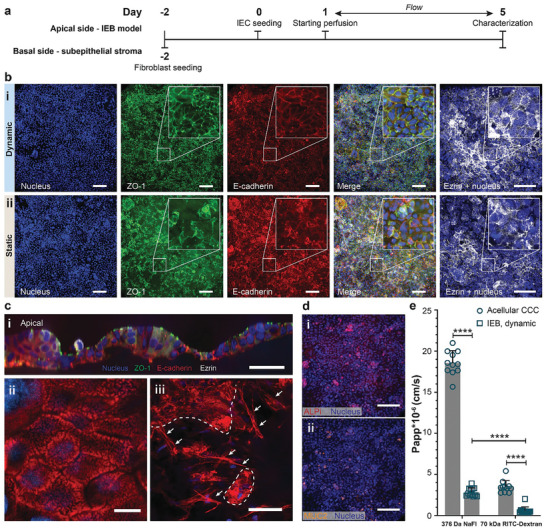
a) Experimental timeline for the formation of the IEB model on chip. b) Characterization of on‐chip IEBs formed under (i) static and (ii) dynamic culture conditions for 5 d. The IEB model with well‐established E‐cadherin and ZO‐1 networks was obtained only under dynamic culture conditions, as shown by z‐projected confocal images of 40–50 µm‐thick z‐stacks. Scale bars: 100 µm. c‐i) A z‐cross section of a polarized IEB model – formed under dynamic culture conditions for 5 d – shows cells with increased height and polarized localization of E‐cadherin and ZO‐1 proteins. Scale bar: 50 µm. ii) Densely packed and well‐defined microvilli, shown by F‐actin staining, were observed on the apical surface of the on‐chip IEB model. Scale bars: 10 µm. iii) Fibroblasts were observed near the IEC layer (marked with white dashed lines). Scale bars: 50 µm. d) IF staining showing the expression of i) enterocyte (ALPi) and (ii) goblet cell markers (membrane‐bound mucus, MUC2). Scale bars: 100 µm. e) Permeability of the IEB model to two fluorescence tracers on day 5, represented as Papp values (n  =  12 basal compartments of 3 individual IEB models). Given values include means ± standard deviation (SD). Statistical analysis by two‐way ANOVA with Tukey‐Kramer post‐hoc test (ns: not significant, ****: *p* < 0.0001).

Our IEB model was generated with multiple representative cell types and required sequential cell‐seeding steps to form a functional barrier. We recapitulated not only the IEC layer of the IEB but also its subepithelial stromal niche – which consisted of stromal cells and ECM – as intestinal stromal cells, particularly fibroblasts, have been shown to actively influence the homeostasis of the IEB and intestine‐resident immune cells in vivo.^[^ ^45^, ^46^ ^]^ To establish the subepithelial stromal niche of the IEB model, a thin coating of ECM protein‐rich basement membrane extract was first deposited on the apical face of the CCC. This basement membrane extract was obtained from an in vitro coculture of human fibroblasts and human epithelial cells and contained collagens, laminin, fibronectin, tenascin, elastin, a number of proteoglycans and glycosaminoglycans, and in vivo levels of growth factors. Second, colonic fibroblasts (CCD‐18Co) were seeded on the basal side of the IEB two days before IEC loading (**Figure** **3**a). As shown in Figure S4a (Supporting Information), fibroblasts not only migrated into the CCC mass but also deposited de novo ECM proteins, such as collagen IV and laminin (Figure S4b, Supporting Information), which reflected their function within the intestinal interstitial matrix in vivo.^[^ ^47^ ^]^

On day 0 of the experiment, IECs (9:1 mixture of Caco‐2 (clone C2BBe1) and HT29‐MTX E12 cell lines) were loaded into the apical compartments, and the microfluidic chip was flipped and kept in an inverted position for 4 h to allow IECs to sediment and attach to the bottom surface of the CCC. Of note, we chose Caco‐2 C2BBe1 cells for our IEB model, as their morphological polarity is homogeneous and comparable to that of enterocytes of the human intestine.^[^ ^48^ ^]^ Similarly, the HT29‐MTX E12 cell line was chosen, based on their ability to produce membrane‐bound mucus – a key characteristic of intestinal goblet cells.^[^ ^49^ ^]^ In our initial tests, the ECM proteins within the basement membrane extract coating promoted firm adhesion of the IECs to the CCC.^[^ ^47^, ^50^ ^]^ After the 4‐hour incubation, nonadherent and dead cells were removed from the apical compartments by flushing of the channel before the chip was filled with medium following an asymmetric culture protocol (Experimental Section, Section 4.3). Gravity‐driven flow was induced in the apical compartments one day after IEC seeding.

#### 2.2.2 On‐chip Maturation and Characterization of the IEB Model

We used Calcein AM staining as a viability indicator to show that our system supported the formation of a viable and confluent cell layer within 2 to 3 d after IEC seeding (Figure S5a, Supporting Information). Counterstaining for Caspase3/7 showed very few apoptotic cells within the IEC layer. From day 3 after IEC seeding, serum was removed from the apical compartments to promote cellular differentiation and barrier polarization (Figure 3a).^[^ ^51^ ^]^ We also characterized the impact of perfusion on the apical side of the IEB model in comparison to static control experiments. Static controls were performed by maintaining MultiU‐Int chips in horizontal positions without perfusion throughout the experimental period. As shown in Figure 3b‐i, on day 5 after IEC seeding, we obtained confluent and polarized IEC layers in the MultiU‐Int chips. Immunofluorescence (IF) staining showed that, under dynamic culture conditions, IECs within on‐chip IEB models expressed a higher degree of membrane localization for the cell adhesion‐junction protein E‐cadherin, the intercellular tight junction protein ZO‐1, and the brush border Ezrin, as compared to cells of IEB models formed under static culture conditions (Figure 3b‐ii). A cross section of a perfused IEC layer showed IECs with increased cell height (in opposed to thin IEC layer obtained under static culture condition as shown in Figure S6a of the Supporting Information), apically localized ZO‐1 and Ezrin, basolaterally localized E‐cadherin (Figure 3c‐i; Figure S6b,c, Supporting Information), and densely packed microvilli on the apical cell surface (Figure 3c‐ii). In the subepithelial stroma of perfused IEB model, fibroblasts dispersed throughout the entire thickness of the CCC, and were also found in proximity to the IECs (Figure 3c‐iii). Altogether, these features confirmed the physiological cellular organization and polarization of the on‐chip IEB model (Video S1, Supporting Information). Further characterization of the perfused IEC layer revealed the expression of differentiation makers of enterocytes (intestinal alkaline phosphatase (ALPi), Figure 3d‐i) and of goblet cells (mucin‐2 (MUC2), Figure 3d‐ii). The alcian blue and periodic acid Schiff staining, which showed a layer of mucins covering the IEB model, further demonstrated the functionality of goblet cells within the IEB model (Figure S5b, Supporting Information). Our data demonstrated that – in our system – proper IEC polarity was attained only under dynamic culture conditions, which evidences the importance of perfusion for establishing a functional on‐chip IEB model. Dynamic culture conditions were crucial for the subsequent experiments involving immune cells, as IEC polarity is a critical factor in both i) vectorial transport of substances across the IEB and ii) mediation of innate immune responses in the subepithelial stromal niche.^[^ ^52^ ^]^

Another key feature of an in vivo IEB is its selective, paracellular permeability that allows for transport of certain molecules from the intestinal lumen to the subepithelial tissue.^[^ ^53^ ^]^ The functionality and integrity of our on‐chip IEB model were, therefore, characterized by measuring the apparent paracellular permeability (Papp) of two different fluorescence tracers – sodium fluorescein salt (NaFl, 376.25 Da) and 70 kDa Rhodamine B‐isothiocyanate‐labeled dextran (RITC‐dextran) – across the barrier. Among these two tracers, NaFl resembled small‐molecule compounds in size and was expected to cross the IEB more freely than the 70 kDa RITC‐dextran. Indeed, as shown in Figure 3e, the Papp value of NaFl was 4.8‐fold higher than that of 70 kDa RITC‐dextran. All Papp values were low and comparable to those reported for other intestine‐on‐chip systems using tracers in the same range of molecular weights.^[^ ^54^, ^55^, ^56^, ^57^ ^]^ Interestingly, acellular CCC specifically hindered the apical‐basal passage of 70 kDa RITC‐dextran, suggesting that CCC also acted as a size‐sensitive barrier and influenced the passive transport of macromolecules across the barrier. The same phenomenon was reported for several types of ECM networks in vivo.^[^ ^58^ ^]^

Overall, we demonstrated that our MultiU‐Int microfluidic system was suitable for the establishment of multiple tight, polarized, and functional in vitro IEB models, which matured on an in vivo‐like stromal niche in less than a week.

### 2.3 MultiU‐Int Microfluidic Chip – A Versatile Tool to Examine the Roles of Different Immune Cell Populations in the Pathogenesis of Inflammatory Bowel Disease and to Perform Therapy Testing

#### 2.3.1 LPS Stimulation of the IEB Model

On its apical side, the in vivo IEB accommodates and constantly interacts with a variety of bacteria – the majority of which are harmless and well tolerated by IECs and the intestinal immune system. To detect microbial invaders, IECs and innate immune cells express different germline‐encoded pattern‐recognition receptors (PRRs), particularly the toll‐like receptor 4 (TLR4), which recognize highly conserved bacterial components known as microbe‐ or pathogen‐associated molecular patterns (MAMPs or PAMPs).^[^ ^59^, ^60^ ^]^ TLR4 specifically recognizes LPS in the cell wall of Gram‐negative bacteria and is capable of orchestrating the balance between tolerogenic and inflammatory responses of intestinal innate immunity.^[^ ^60^ ^]^

**Figure 4:**
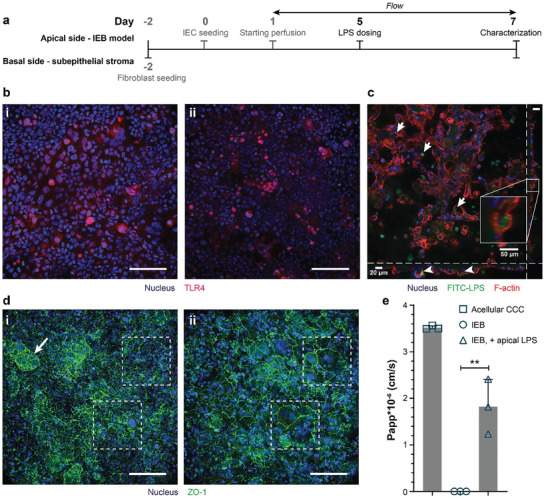
a) Experimental timeline showing the time point and site of LPS administration. b) TLR4 expression in IECs on day 7 i) without and ii) with LPS exposure (10 ng mL^−1^ LPS), shown by z‐stack projection. Scale bars: 100 µm. c) Internalization of FITC‐labeled LPS by IECs (arrows) shown by xy‐ (largest insert), xz‐, and yz‐ cross‐sections of a z‐stack. Cell borders were visualized by F‐actin staining. Scale bars: 50 µm. d) IF staining of the IEB model at day 7 under different conditions: i) control IEB model without apical LPS with 3D cellular organization (arrow) and well‐established ZO‐1, ii) IEB with 10 ng mL^−1^ LPS on the apical side. The LPS‐treated IEB model was underdeveloped in 3D, and giant, multinuclear cells were observed within the barrier while IECs within the control barrier appeared to have small and even cell size (dashes boxes). Scale bars: 100 µm. e) IEB model permeability for 70 kDa RITC‐dextran at day 7, shown as Papp values (*n*  =  3 basal compartments of 3 individual IEB models). Values are means ± SD. Statistical analysis by one‐way ANOVA with Tukey–Kramer post hoc test (**: *p* < 0.01).

Recent research has shown that, in addition to mutual interactions between the microflora and the IECs, the balance within the microflora plays an important role in maintaining intestinal homeostasis.^[^ ^61^, ^62^, ^63^, ^64^, ^65^ ^]^ A decrease in microbial diversity – together with the enrichment of pathogenic bacteria (e.g., *Escherichia coli*) within the intestinal microflora – have been linked to the immunopathogenesis of IBD.^[^ ^66^ ^]^ To mimic such microbial imbalance, especially the enrichment of pathogenic bacteria on the apical side of the in vivo IEB, we exposed the apical side of our on‐chip IEB model to 10 ng mL^−1^ of LPS, which was derived from a highly toxic *E. coli* bacterial serotype, for 2 d (day 5 to day 7 post‐IEC seeding; **Figure** **4**a).^[^ ^67^ ^]^

As shown in Figure 4b‐i, the IECs within our on‐chip IEB model expressed TLR4, suggesting a potential interaction with LPS. Notably, the level of TLR4 expressed by IECs within our cell line‐based IEB exhibited no significant change rather than an increase upon LPS (endotoxin) challenge^[^ ^68^ ^]^ (Figures 4b‐ii and S7a, Supporting Information). In fact, TLR4 expression in Caco‐2 cells has been reported to remain unaffected by LPS.^[^ ^69^ ^]^ Nonetheless, in our system, LPS uptake by IECs was evidenced when fluorescein isothiocyanate (FITC)‐labeled LPS of the same bacterial serotype was used (Figure 4c). Additionally, we observed an LPS‐dependent increase in the expression of the autophagy marker LC3, manifested as large puncta shown in Figure S7b (Supporting Information), further confirming the response of IECs to the presence of LPS.

As shown in Figure 4d‐i, on day 7, the IEC layer of control IEB models (without apical LPS exposure) developed a more defined 3D morphology and exhibited more robust expression of ZO‐1 and Ezrin proteins. Additionally, the cell size was small and quite even. In contrast, when the IEB model was apically exposed to LPS, the IEC layer appeared to be flatter (Figure 4d‐ii), yet remained well polarized. We observed several giant, multinucleated cells within the IEB model (Figure 4d‐ii) only when LPS was present in the apical compartment. We postulated that these changes in IEC morphology were part of their defending mechanism in response to the highly toxic LPS serotype that has been used to stimulate the on‐chip IEB model.^[^ ^70^, ^71^ ^]^ Additionally, as shown in Figure 4e, apical exposure of the IEB model to LPS led to an increased permeability of the barrier to 70 kDa RITC‐dextran. These results are in line with those of previous in vitro and in vivo studies that showed LPS‐induced disruption of the IEB.^[^ ^72^, ^73^, ^74^, ^75^ ^]^

#### 2.3.2 Versatile Experimental Setups on the MultiU‐Int Microfluidic Chip

**Figure 5:**
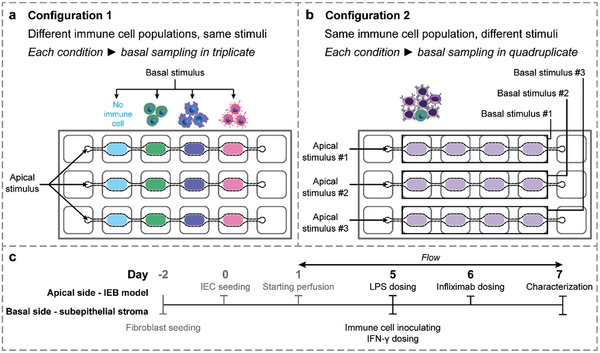
Versatile experimental setup using the MultiU‐Int microfluidic chip. a) Different immune cell populations (i.e., MNs, MFs, and iDCs) were co‐cultured with a single on‐chip IEB model at separate locations, allowing for side‐by‐side comparison of cell type‐specific responses to the same stimuli. b) Similar IEB model‐immune cell co‐cultures were exposed to different stimuli on the same chip, allowing for the scrutiny of distinct cellular behaviors in response to different stimuli. c) Experimental timeline showing the time point and site of immune cell loading, triggering of inflammation, and therapy administration.

To showcase the versatility of our system, we conducted proof‐of‐concept experiments with two different configurations, as illustrated in **Figure** **5**. In the first configuration (experimental configuration 1, Figure 5a), each on‐chip IEB model was co‐cultured with distinct subsets of MNPs, namely monocytes (MNs), MN‐derived macrophages (MFs), and MN‐derived immature dendritic cells (iDCs), at separate basal locations. All co‐cultures on the same MultiU‐Int chip were exposed to the same inflammatory stimuli. This configuration allowed for observing the inflammatory responses of these distinct MNP subsets side by side. In the second configuration (experimental configuration 2, Figure 5b), all three IEB models on the MultiU Int chip were co‐cultured with the same mixed population of immune cells – PBMCs – in all basal compartments. However, each model was exposed to different combinations of inflammatory stimuli. This configuration enabled a direct comparison of how the same immune cell population responded to different stimuli.

For both configurations, to trigger the inflammatory responses, IFN‐γ (20 ng mL^−1^) was administered to the basal compartments of the IEB model‐immune cell co‐cultures^[^ ^41^ ^]^ on day 5 of the experiment (Figure 5c). IFN‐γ is mainly produced by innate lymphoid cells (ILCs) – a heterogeneous group of cytokine‐producing lymphocytes that is predominantly located at mucosal barrier surfaces, such as the IEB.^[^ ^76^ ^]^ The proximity of ILCs to the IEB enables them to sense microbial endotoxin or tissue damage at an early stage and to produce IFN‐γ to recruit other innate immune cells, such as MNPs.^[^ ^38^, ^77^, ^78^ ^]^ IFN‐γ is also an important cytokine for immune responses by CD4^+^ cells^[^ ^76^ ^]^ and has been shown to drive excessive immune responses against microbial components within the apical content in IBD.^[^ ^77^ ^]^

As an increased release of TNF‐α is one of the hallmarks of intestinal inflammation, we used an anti‐TNF‐α therapeutic antibody – Infliximab – to demonstrate testing of anti‐inflammatory therapies with our chip system. The anti‐inflammatory antibody Infliximab was added to basal compartments of selected inflamed IEB models on day 6 of the experiment, emulating its intravenous administration in clinical settings. As our microfluidic chip allowed for direct access to the basal compartments of the IEB, sampling of cells and cell culture supernatant could be carried out during the experiment. We used i) different flow cytometry staining panels to analyze cell identities and composition and ii) chemokine/cytokine (chemo/cytokine) profiling to assess subset‐specific inflammatory responses.

*Experimental configuration 1*: In the early stages of IBD research, IBD was believed to be driven solely by adaptive immunity. However, recent studies have shown that innate immunity plays an equally important role in initiating and sustaining IBD.^[^ ^78^, ^79^ ^]^ Inappropriate activation of MNPs, such as MNs, MFs, and iDCs, has been shown to underly intestinal dyshomeostasis, chronic inflammation, and IEB damage, all of which are hallmarks of IBD.^[^ ^11^, ^80^ ^]^

MNPs can be activated by LPS through binding of LPS to LPS‐receptor complexes including TLR4 on the cell surface. Downstream of this LPS recognition is the recruitment of particular intracellular adaptor protein complexes that i) activate or induce MNP maturation and ii) induce the expression of inflammatory markers (i.e., CD64) and inflammatory cytokines (i.e., TNF‐α, granulocyte‐macrophage colony‐stimulating factor (GM‐CSF), interleukin‐6 (IL‐6), and IL‐1β).^[^ ^81^, ^82^, ^83^ ^]^

For the first proof‐of‐concept experiment, we inoculated each basal compartment of an on‐chip IEB model with a different THP‐1 MN‐derived MNP subset on day 5 after IEC seeding (Figure 5a). In each IEB model, one basal compartment remained free of MNPs and was used as a control. All the three IEB models on the same MultiU‐Int chip were exposed to the same combination of apical and basal inflammatory stimuli, hence, different chips were used to test different combinations of apical and basal inflammatory stimuli. Detailed characterizations of MN‐derived MNP subsets are described in the Supplementary materials and methods (Section 1) and Figure S8 (Supporting Information).

**Figure 6:**
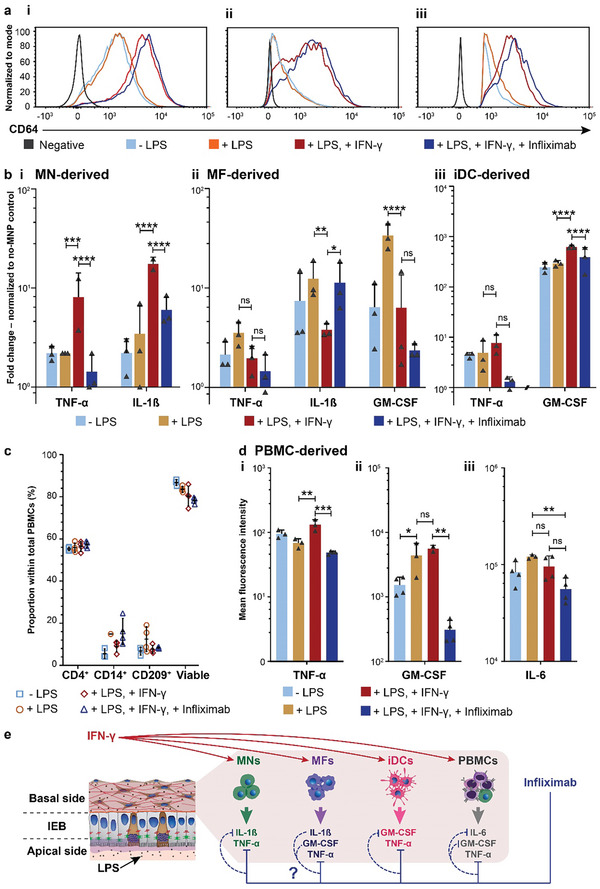
a) Histogram plot showing relative changes in CD64 expression of i) MNs, ii) MFs, and iii) iDCs in on‐chip IEB model‐MNP co‐cultures. b‐i) MN‐derived, ii) MF‐derived, and iii) iDC‐derived cytokine profiles at day 7 (*n*  =  3). Data is represented as x‐fold change with respect to the baseline of the “no‐MNP” control. Data was acquired from three individual basal compartments (*n* = 3) of one representative experiment out of a total of three independent experiments. c) Cell compositions (shown as the proportion of cells that were positive for CD4, CD14, and CD209, respectively) within PBMC populations in on‐chip IEB model‐PBMC co‐cultures under different conditions (*n*  =  4). Data was acquired from four individual basal compartments of the same IEB model (*n* = 4). d) Changes in levels of representative cytokines (TNF‐ α, GM‐CSF, and IL‐6) detected in on‐chip IEB model‐PBMC co‐cultures (*n*  =  4). Values shown in b) and d) are mean ± SD and statistically compared by two‐way and one‐way ANOVA with Tukey‐Kramer post‐hoc test, respectively (ns: not significant, *: *p* < 0.05, **: *p* < 0.01, ***: *p* < 0.001, and ****: *p* < 0.0001). e) Schematic illustration summarizing key inflammatory chemo/cytokine signaling of different immune cell subsets on chip and the proposed mechanism of action of Infliximab (Solid lines indicate direct effects, while dashed lines indicate an indirect effect of Infliximab.).

To assess the maturation and activation of MN‐derived MNP subsets, we analyzed multiple relevant cell surface markers (Figures S9a,S10a, and S11a, Supporting Information), particularly the myeloid cell activation marker CD64 (**Figure** **6**a). In the absence of inflammatory stimuli, cell maturation was evident for all MNP subsets after 2 d in on‐chip co‐cultures as shown by an increase in CD64^+^ populations: MNs (from 9% to 47–54%, Figures 6a–i and S9a, Supporting Information), MFs (from 7% to 42–39%, Figures 6a–ii and S10a, Supporting Information), and iDCs (from 13% to 18‐74%, Figures 6a–iii and S11a, Supporting Information). Among the three subsets, only iDCs showed LPS‐dependent expression of CD64, possibly related to their ability to directly sample the apical content for early detection of antigens, in this case LPS.^[^ ^84^, ^85^ ^]^ IFN‐γ treatment significantly increased CD64^+^ populations for all subsets, indicating a shift to an inflammatory phenotype.^[^ ^86^, ^87^, ^88^ ^]^ In addition to acquiring the CD64^+^ pro‐inflammatory M1 phenotype, IFN‐γ‐treated MFs also polarized into the CD163^+^ anti‐inflammatory M2‐like phenotype, albeit at a lower proportion (10% of the single‐cell MF population) (Figure S10a, Supporting Information). More iDCs matured and were activated under LPS‐ and IFN‐γ‐dependent inflammatory conditions (Figure S11a, Supporting Information).

Upon additional Infliximab treatment, the CD64^+^ population slightly fluctuated in all subsets. Furthermore, as shown in Figure S9a (Supporting Information), Infliximab treatment led to a 10% decrease in the CD14^+^ MN population. A similar decrease in CD14 expression, caused by Infliximab therapy, has been i) associated with a decrease in MN activation in UC patients and ii) considered one of the early markers of a positive therapeutic response.^[^ ^89^ ^]^ Detailed descriptions of the gating strategy, changes in cell surface markers, and their co‐expression for each subset are shown in the Supplementary materials and methods (Section 2) and Figures S12,S13, and S14 (Supporting Information), respectively.

The existence of an inflammatory milieu in all IEB model‐MNP co‐cultures upon LPS and IFN‐γ co‐administration was further confirmed by their chemo/cytokine profiles (Figures 6b and S9,S10, and S11, Supporting Information, subfigures b and c). As shown in Figure 6b‐i, TNF‐α, and TNF‐α‐inducible IL‐1β levels increased drastically in inflamed IEB model‐MN co‐cultures, whereas increased TNF‐α levels were accompanied by high levels of GM‐CSF in inflamed IEB model‐iDC co‐cultures (Figure 6b‐iii). Upregulation of GM‐CSF production by iDCs in response to the invasion of pathogenic bacteria was reported in animal models,^[^ ^90^ ^]^ suggesting a proper inflammatory response by iDCs in our system. Interestingly, in the IEB model‐MF co‐cultures, apical dosing with LPS alone resulted in high levels of GM‐CSF, which decreased upon LPS and IFN‐γ co‐administration (Figure 6b‐ii). We hypothesize that i) high levels of GM‐CSF, released upon LPS stimulation, and ii) the emergence of anti‐inflammatory M2 MF may be responsible for the decreased expression of pro‐inflammatory cytokines. Although GM‐CSF has been commonly considered as pro‐inflammatory cytokine, recent studies have shown that in IBD, it can downregulate inflammatory responses as a part of a complex signaling cascade to restrain IBD pathogenesis.^[^ ^91^, ^92^, ^93^, ^94^ ^]^

As expected, Infliximab administration effectively neutralized TNF‐α and TNF‐α‐inducible IL‐1β and GM‐CSF secreted by MNs and iDCs to unstimulated levels. Although neutralization by Infliximab was observed with MF‐derived cytokines, the effect was not as profound as that of MN‐ and iDC‐derived cytokines.

*Experimental configuration 2*: In the second experimental setup (Figure 5b), naïve PBMCs were seeded into all basal compartments of the MultiU‐Int microfluidic chip, and three individual on‐chip IEB models were exposed to different combinations of apical and basal stimuli on day 5: i) apical LPS only, ii) apical LPS and basal IFN‐γ, and iii) apical LPS and basal IFN‐γ, followed by one day of Infliximab treatment. As shown in Figure 6c, in the on‐chip co‐cultures, CD4^+^ T cells – the main T cell population found within the intestinal subepithelial stroma^[^ ^88^ ^]^ – represented ≈55% of the total PBMCs, while less than 15% of the cells were CD14^+^. These cell compositions remained unchanged for the basal administration of IFN‐γ alone or in combination with Infliximab.

In most cases, we were able to retrieve ≈25% of the inoculated immune cells from each basal compartment for flow‐cytometry analysis, as immune cells did migrate into the collagen I membrane during co‐culture experiments (Videos S2 and S3, Supporting Information). The retrieval rate varied for different immune cell populations. Cell retrieval rate was higher for PBMCs (ranging from 25% to almost half of the inoculated cells), followed by monocytes, dendritic cells, and macrophages. This observation reflects the tendency of the cells to adhere to surfaces, with macrophages being the only cell type exhibiting adherent morphology (Figure S7b–i, Supporting Information).

Despite such unchanged cell compositions, we measured increased levels of TNF‐α (Figure 6d–i) when the IEB model‐PBMC co‐culture was co‐stimulated with LPS and IFN‐γ, indicating an ongoing inflammation. Substantial levels of GM‐CSF and IL‐6, which remained unchanged upon IFN‐γ administration, were detected in IEB model‐PBMC cocultures (Figure 6d–ii,iii). Similar to what was observed in the IEB model‐MNP cocultures, Infliximab administration effectively neutralized TNF‐α and resulted in decreased levels of GM‐CSF and IL‐6. The complete chemo/cytokine profiles of the IEB model‐PBMC co‐cultures are shown in Figure S15 (Supporting Information). Figure 6e depicts key inflammatory chemo/cytokine signaling of different immune cell subsets on chip and the proposed mechanism of action of Infliximab.

#### 2.3.3 Alteration of On‐chip IEB Models in Response to Inflammation

As part of our proof‐of‐concept experiment, we measured the changes in the permeability of IEB models in co‐cultures with MNPs. As shown in Figure S16 (Supporting Information), under control conditions (no‐LPS, no‐MNP), the tightness of the IEB models further increased from day 5 to day 7, as indicated by the decreased Papp values of 70 kDa RITC‐dextran. Although the Papp values increased in cocultures of the IEB model and MNPs, they remained very low – well below the values of the acellular CCC. We also observed well‐established ZO‐1 networks in the IEB model‐MNP co‐cultures (Figure S17a, Supporting Information). Apical administration of LPS led to an increase in barrier permeability only when MNPs were not included on the basal side of IEB model (Figures 4e and S16, Supporting Information). MNPs seemed to avert the negative effect of LPS on the integrity of IEB models, as the Papp values for the LPS‐treated IEB model‐MNP co‐cultures remained low and comparable to those of the controls without LPS and MNP. Similarly, IFN‐γ appeared to exert its protective role on IEB models when MNPs were not present, as implied by low Papp values. Indeed, the contribution of MNPs and cytokines, such as IFN‐γ, in maintaining IEB integrity through various soluble factors has been reported previously but is incompletely understood.^[^ ^95^, ^96^, ^97^, ^98^ ^]^ For all IEB model‐MNP co‐cultures, IFN‐γ did not induce any changes in the permeability of 70 kDa RITC‐dextran (Figure S16, Supporting Information), although IF staining revealed downregulation and slight disruption of the ZO‐1 network (Figure S17b, Supporting Information). These morphological observations suggested that LPS‐induced damage of the IEB only increased when immune components (i.e., IFN‐γ and MNPs) were absent.

#### 2.3.4 On‐chip Extracellular Matrix Remodeling

Remodeling of the ECM within the interstitial matrix has recently gained attention as a pathological feature of IBD.^[^ ^99^ ^]^ In addition to studies on immunopathogenesis, our MultiU‐Int microfluidic chip offers the potential to investigate structural changes of the ECM during IBD progression. To assess such changes, we optically monitored ECM proteins, such as collagen I, and measured the secretion of remodeling enzymes, such as matrix metalloprotease 1 (MMP‐1). As shown in Figure S18a (Supporting Information), collagen I fibers in an acellular CCC appeared as thick bundles and were mostly aligned in a horizontal orientation with respect to the cell culture interfaces. During extended culturing periods of up to 9 days without any inflammatory stimuli, we observed a reduction in second‐harmonic‐generation (SHG) signal by collagen I in the cocultures of fibroblasts, the IEB model, and immune cells (Figure S18b‐i). Such reduction in SHG signal may be caused by fibroblasts’ and MNPs’ i) active degradation or ii) reorganization in the structure and angle orientation of collagen I fibers within the CCC.^[^ ^100^, ^101^ ^]^ Apical dosing of LPS resulted in the formation of thick bundles of collagen I, which appeared to be more pronounced in comparison to acellular CCC (Figure S18b‐ii). Basal administration of IFN‐γ induced reorganization of collagen I fibers into a higher degree of alignment in cocultures of IEB model and MNs or MFs (Figure S18b‐iii). Detection of MMP‐1 in the supernatant, collected from basal compartments of all coculture conditions (Table S1 and Figure S19, Supporting Information), further suggested an active ECM remodeling on‐chip.

## 3 Discussion

In this work, we present a novel intestine‐on‐chip system that was designed to recapitulate and decipher the immunopathogenesis of IBD. Our approach approximates a realistic recapitulation of an in vivo IEB and its adjacent components within the intestinal subepithelial tissue (Figure 1). In our proof‐of‐concept study, the on‐chip IEB model was formed by combining cell lines representing the most abundant cell types within the native IEB – enterocytes and mucus‐producing goblet cells. Furthermore, we included the colonic fibroblasts in the basal compartments for forming the IEB model, as fibroblasts have been proven to support the development, to maintain tissue homeostasis, and to regulate the function of the IEB.^[^ ^45^, ^102^, ^103^ ^]^ In contrast to previously published approaches, for which rigid, plastic‐based porous membranes were used to provide physical support for the IEB model, we formed our IEB model on an elastic and fibrous membrane made from native collagen I fibers (shown as CCC in Figure 2). As the collagen membrane is sensitive to high temperatures and difficult to bond in a microfluidic system, a room‐temperature assembly protocol and auxiliary layers have been used to integrate this layer into the microfluidic chip. A thin layer of ECM‐protein‐rich basement membrane extract was deposited at the membrane‐IEC interface to mimic the surface of the intestinal basement membrane.^[^ ^102^ ^]^ This ECM‐coated CCC resembled the elastic in vivo subepithelial stroma, in which intestinal fibroblasts (Figure S4a, Supporting Information) and immune cells (Videos S2 and S3, Supporting Information) reside.^[^ ^47^ ^]^ Moreover, unlike inert plastic membranes, which are solely used as a substrate for cell attachment, the CCC (with a thickness of ≈35 µm to 45 µm)^[^ ^104^ ^]^ enabled cell migration and bidirectional interaction with the IECs and stromal cells. This feature shows the potential of the system for investigations of other IBD‐associated complications, such as fibrosis. We showed that ECM‐cell and cell‐cell interactions were evident in our system, as the fibroblasts migrated into the collagen I membrane and resided in proximity to the IEC layer (Figures 3c‐iii and S4a, Supporting Information), forming a physiologically relevant subepithelial stromal niche.

The dense network of collagen I fibers within the CCC also acted as a size‐selective barrier for the apical‐basal passage of substances as shown in Figure 3e. This observation was in line with the selective permeability of an in vivo basement membrane and its adjoining interstitial matrix,^[^ ^47^ ^]^ an important feature that is often neglected in in vitro studies. This feature of the CCC also helped to separate the signaling milieus between basal compartments by hinder the diffusion of released chemo/cytokines as the chemo/cytokines of interest possess rather high molecular weights (e.g., IFN‐γ: 15‐25 kDa,^[^ ^105^ ^]^ GM‐CSF: 14‐35 kDa^[^ ^106^ ^]^).

Shear stress – which is critical for the development of in vitro IEB models^[^ ^21^ ^]^ – was induced by gravity‐driven flow. We proved with IF staining, permeability assays (Figure 3), and mucus staining (Figure S5b, Supporting Information) that an array of tight, uniformly polarized, and functional in vitro IEB model units was simultaneously formed in our MultiU‐Int microfluidic system within 7 d in a compact, user‐friendly experimental setup. The morphology, tightness, and formation time of our on‐chip IEB model were comparable to those of other intestine‐on‐chip models.^[^ ^21^, ^22^, ^23^ ^]^

In the next step, we demonstrated that this novel intestine‐on‐chip system can support different experimental approaches to investigate different aspects of IBD pathogenesis. To mechanistically investigate the inflammatory responses, we introduced LPS to the apical compartment of our IEB model instead of using living bacteria. We first administered LPS to the apical side of the on‐chip IEB model to simulate physiological exposure to Gram‐negative‐bacteria‐derived endotoxins in the in vivo intestinal lumen. Morphologically, LPS negatively affected the 3D organization of the on‐chip IEB, and the LPS‐exposed IEB featured several giant and multinucleated cells (Figure 4d‐ii). Furthermore, LPS induced a decrease in barrier integrity as shown by increased permeability of the IEB to RITC‐dextran (Figure 4e). We attributed these changes to LPS‐induced downregulation and redistribution of tight‐junction proteins that eventually loosened up the intercellular tight junctions and enabled apical contents to enter the subepithelial stromal niche.^[^ ^72^, ^107^ ^]^ In agreement with previous studies, we also showed that IECs within the IEB model expressed TLR4 and were able to internalize LPS (Figure 4c), implying that they may actively respond to LPS exposure.^[^ ^108^ ^]^

To investigate the inflammatory responses within the MultiU‐Int microfluidic chip, in addition to LPS exposure, we i) inoculated the basal compartments of on‐chip IEB models with different immune cell populations (MNP subsets or PBMCs) and ii) administered the inflammatory cytokine IFN‐γ to specific basal compartments, using two different experimental configurations (Figure 5). In both cases, the role of IFN‐γ as a trigger of inflammation was confirmed by analyzing changes in the expression of surface markers of the included immune cells (Figure 6a,c, S9a, S10a, and S11a, subfigures (i)) and the on‐chip chemo/cytokine repertoires (Figures 6b,d and S9, S10, and S11b,c, Supporting Information). While no obvious change in cell surface markers was observed for PBMCs (Figure 6c), an increased expression of the myeloid cell activation marker CD64 in all MNP subsets evidenced an ongoing inflammation (Figure 6a).^[^ ^88^, ^109^ ^]^ The spatially‐separated arrangement of the basal compartments allowed for individual sampling and measurement of distinct chemo/cytokine responses of each immune cell type, even when these cells shared a single IEB model as in the case of MNPs. Inflammatory responses by MNs featured increased levels of TNF‐α and IL‐1β (Figure 6b‐i) that were triggered only by co‐stimulation with LPS and IFN‐γ. This finding is in agreement with clinical studies suggesting that MN‐derived TNF‐α and IL‐1β are high‐level drivers of intestinal inflammation in IBD.^[^ ^110^, ^111^ ^]^ We showed that iDCs properly matured, underwent activation, and exhibited apical antigen sampling functions, as inflammatory stimuli induced higher levels of GM‐CSF in IEB model‐iDC co‐cultures (Figure 6b‐iii). Such proper maturation and activation of iDCs has been shown to initiate subsequent T cell‐driven inflammatory responses in the onset of IBD.^[^ ^112^ ^]^ We also observed that LPS alone was sufficient to induce an elevated level of GM‐CSF in IEB model‐MF co‐cultures (Figure 6b‐ii), suggesting that, besides iDCs, MFs were also capable of actively sampling the apical content.^[^ ^113^ ^]^ Videos S2 and S3 (Supporting Information) show that activated (NF‐κB^+^) iDCs and MFs were detected in the immediate vicinity of the IEC layer. Interestingly, we measured decreased levels of MF‐derived GM‐CSF when an inflammation was triggered by IFN‐γ. Although GM‐CSF is mostly associated with pathogenic inflammation, it is a pleiotropic cytokine, the anti‐inflammatory and protective function of which has been increasingly appreciated. Although clinical studies have shown conflicting results concerning the role of GM‐CSF in IBD, diminished levels of GM‐CSF were shown to be associated with increased severity of Crohn's disease and higher susceptibility to acute DSS‐induced colitis in experimental mouse models.^[^ ^92^, ^114^, ^115^ ^]^

Experiments with PBMCs revealed that the combination of LPS and IFN‐γ also successfully induced inflammatory responses of PBMCs as indicated by elevated levels of TNF‐α (Figure 6d‐i). We assumed that the increased levels of TNF‐α came from both CD14^+^‐myeloid cells and CD4^+^‐T cells, as IFN‐γ is known to evoke Th1‐type immune responses by CD4^+^ T cells.^[^ ^116^ ^]^ We also observed different cytokine expression patterns of THP‐1‐derived cells and PBMCs following inflammatory induction. In our system, THP‐1‐derived cells produced modest levels of IL‐6 that remained unchanged regardless of the inflammatory stimuli (Figures S9c, S10c, and S11c, Supporting Information). In contrast, PBMCs produced substantial amounts of IL‐6 in co‐culture with IEB models (Figure 6d‐iii), the largest fraction of which may have been produced by CD14^+^‐myeloid cells and CD4^+^‐T cells.^[^ ^117^ ^]^

In this proof‐of‐concept work, we demonstrated that our system allowed for polarized dosing of compounds to the developed IEB model. We selected Infliximab – the most commonly used TNF blocker for IBD treatment – and mimicked its intravenous administration by dosing the compound to the basal compartments of the on‐chip IEB model. Infliximab attenuates intestinal inflammation by neutralizing the biological activity of soluble TNF‐α and initiating other TNF neutralization‐independent anti‐inflammatory mechanisms, which are only incompletely understood.^[^ ^118^ ^]^ We successfully recapitulated the neutralizing effect of TNF‐α by Infliximab in our system, as the levels of TNF‐α, detected in the basal compartments of all cocultures, decreased to variable extents with Infliximab administration. Infliximab treatment also led to a decreased release of IL‐1β (in IEB model‐MN co‐cultures) and GM‐CSF (in IEB model‐MF, IEB model‐iDC, and IEB model‐PBMC cocultures), as the production of both pro‐inflammatory cytokines was stimulated by TNF‐α.^[^ ^119^, ^120^ ^]^ Notably, the level of IL‐6 within IEB model/PBMC co‐cultures diminished upon Infliximab administration (Figure 6d‐iii). Indeed – similar to our results – the IL‐6 level was shown to be reduced in IBD remission.^[^ ^121^ ^]^

Our results show that the inclusion of relevant cellular and noncellular components is critical to recapitulate the complex interplay of chemo/cytokine signaling at the IEB interface and to reveal potential contributions to intestinal inflammation. On the one hand, experiments with a single immune cell population, such as different subsets of MNPs, are useful to reveal the specific responses of each immune cell population to inflammatory triggers. On the other hand, the combination of diverse immune cell types (e.g., PBMCs) helps to reveal how innate immunity can induce adaptive immunity imbalance. To this end, the use of patient‐derived IECs together with autologous stromal and immune cells will support mechanistic investigations of what ignites overactive mucosal immune responses given the genetic background of the patient. Moreover, inclusion of intestinal resident immune cells, especially ILCs, in on‐chip IEB models may eliminate the need for external inflammatory triggers, such as IFN‐γ, and help to better recapitulate the intestinal immune microenvironment at the onset of IBD.

In summary, the MultiU‐Int chip system represents a versatile tool to emulate and investigate the IEB, its interaction with different immune cell populations, and intestinal inflammations. We demonstrated that our on‐chip IEB model features physiological architectures and functionality of both, the IEC layer and its adjacent subepithelial tissue. We also showed that the chip system enables investigations of the complex network of chemo/cytokines that are relevant components of the immune response to inflammatory stimuli. As different experimental conditions can be tested on the same IEB model – which can be established with a low initial number of cells – our system is well‐suited for patient‐cell‐based experimentation. The microfluidic system offers parallelization of experiments without the need for complex fluidic pumps or actuators. The accessibility of both sides of the barrier enabled polarized dosing and potentially enables to study the simultaneous application of different therapeutic approaches. Therefore, the system constitutes a promising tool to gain a better understanding of the role of disease‐relevant immune cell types or cytokines, as well as to enable therapy testing and the identification of new therapeutic targets.

Our primary objective included the development of a biologically relevant intestinal barrier model and the use of such a model to study different factors contributing to intestinal inflammation. The resulting system featured significant complexity, as it included interactions among three different cell types within the epithelial barrier and its stroma, interactions of the immune cells with cells of the barrier, and immune responses triggered by inflammatory stimuli. Consequently, the obtained results required careful interpretation, and more extensive characterization of the current system will need to be performed before introducing additional layers of complexity, such as intestinal microbes^[^ ^122^ ^]^ and a primary‐cell‐derived barrier model for obtaining the 3D spatial distribution of epithelial cells.^[^ ^123^ ^]^ Nevertheless, functional barrier models without incorporating 3D villi‐crypt configurations have been developed by other groups and successfully been used to address various biological questions.^[^ ^124^, ^125^, ^126^, ^127^ ^]^ In future efforts, periphery instruments will be developed and integrated to the system to realize different aerobic/anaerobic conditions and to enable the integration of microbiota in our system. Such integration will enable to selectively maintain anaerobic conditions in the apical compartments of the barrier models and to establish a more physiologically relevant oxygen gradient across different layers of the barrier model.

In its current state, experiments with the system feature a rather low temporal resolution, which can, however, be markedly improved by (i) integration of on‐line sensors to monitor trans‐epithelial electrical resistance, oxygen, or cytokine concentrations into the microfluidic chip or (ii) application of an automatic tilting and imaging system. These additional features will enable detailed investigations of the dynamics of cell‐cell interactions, of the emergence of pathogenic morphologies, and of inflammatory signaling.

## 4 Experimental Section

### Cell Culture

The human colon adenocarcinoma cell line Caco‐2, clone C2BBe1 (ATCC CRL‐2102; American Type Culture Collection (ATCC), Manassas, VA, USA), human colon cell line HT29‐MTX E12 (12 040 401; Merck, Darmstadt, Germany), and colonic fibroblast CCD‐18Co (ATCC CRL‐1459; ATCC) were cultured in their *maintenance medium* – Dulbecco's modified Eagle's medium (DMEM) (ATCC), supplemented with 10% heat‐inactivated fetal bovine serum (h.i. FBS; Gibco, Thermo Fisher Scientific, Waltham, MA, USA), 1× nonessential amino acids (NEAA) (Merck), and 1× Kanamycin sulfate (Gibco, Thermo Fisher Scientific) – using tissue culture‐treated cell culture flasks (Greiner Bio‐One, Kremsmünster, Austria). Medium exchange was performed every 2 d or when signs of acidification of the cell culture medium appeared. The Caco‐2 cell culture was sub‐cultured when reaching ≈50–60% confluence, while HT29‐MTX E12 and CCD‐18Co were sub‐cultured at 80‐85% confluence. The human acute monocytic leukemia cell line THP‐1 NF‐κB‐eGFP was purchased from Merck and cultured in Roswell Park Memorial Institute 1640 medium (RPMI 1640; Gibco, Thermo Fisher Scientific), supplemented with 10% h.i. FBS, 1 × 10^−3^ M sodium pyruvate (Merck), 1 × 10^−3^ M N‐2‐hydroxyethylpiperazine‐N‐2ethane sulfonic acid (Gibco, Thermo Fisher Scientific), 1× Kanamycin sulfate, and 5 × 10^−5^ M 2‐mercaptoethanol (Gibco, Thermo Fisher Scientific) using suspension culture flasks (Greiner Bio‐One). This THP‐1 cell line was stably transfected with an NF‐κB‐eGFP reporter construct, the expression of which can be induced by appropriate stimuli and detected by flow cytometry or fluorescence microscopy. THP‐1 NF‐κB‐eGFP cell cultures received a half medium exchange every 2 to 3 d or when the cell density reached 1 to 1.5 × 10^6^ cells mL^−1^. Naïve human PBMCs were purchased from Lonza (Basel, Switzerland) and cultured in RPMI 1640 medium supplemented with 10% h.i. FBS, 1 × 10^−3^ M sodium pyruvate (Merck), 1× Kanamycin sulfate, and 5 × 10^−5^ M 2‐mercaptoethanol (Gibco, Thermo Fisher Scientific) using Corning Costar ultra‐low attachment well plate (Corning, MA, USA). All cell cultures were maintained in a humidified incubator at 37  °C and 5% CO_2_ (Binder CB 220, Tuttlingen, Germany). All experiments were performed with Caco‐2, HT29‐MTX E12, and CCD‐18Co cells between passages 48 and 65, 53 and 70, and 4 and 15, respectively. THP‐1 NF‐κB‐eGFP cells were used within 15 passages after initial thawing, while naïve PBMCs were used right after thawing. All cells were routinely tested for mycoplasma contamination and found negative.

In this work, we used the monocytic THP‐1 NF‐κB‐eGFP cell line to generate three different types of MNPs: monocytes (MNs), macrophages (MFs), and immature dendritic cells (iDCs). The monocytic THP‐1 NF‐κB‐eGFP cells themselves were used as MNs. To obtain MFs from THP‐1 NF‐κB‐eGFP MNs, we differentiated them using 5 × 10^−9^ m PMA (Merck) in the complete culture medium for 48 h^[^ ^128^ ^]^ at a density of 10^6^ cells mL^−1^. To obtain iDCs, we treated THP‐1 MNs with 250 IU mL^−1^ recombinant human IL‐4 (Peprotech, Thermo Fisher Scientific) and 1000 IU mL^−1^ recombinant GM‐CSF (Gibco, Thermo Fisher Scientific) for 5 d,^[^ ^129^ ^]^ also at a density of 10^6^ cells mL^−1^. A half medium exchange was performed every 2 d for the iDC culture. Tissue culture‐treated well plates (Greiner Bio‐One) were used for culturing all three aforementioned cell types.

### MultiU‐Int Microfluidic Chip Fabrication

The MultiU‐Int microfluidic chip was designed in the size of a standard microscopy slide (25 mm × 75 mm) and included 3 main layers. The bottom layer of the microfluidic chip consisted of 3 parallel microfluidic channels that served as the apical compartments of the on‐chip IEB model. Six spatially separated compartments were located on top of each apical compartment. Among these six compartments, the two outermost compartments served as medium reservoirs that flanked both ends of the apical compartments. The four compartments in the middle served as the basal compartments of the IEB model, where fibroblasts – and at a later stage of the experiment – immune cells were added. The on‐chip IEB model was accommodated in the middle layer of the chip between the apical and basal compartments. We used a commercially available, polystyrene‐based sticky‐slide 18 well part (ibidi, Gräfelfing, Germany) as the top layer of the chip. To form the middle layer of the chip, strips of CCC (branded as Collagen Cell Carrier; Viscofan BioEngineering, Weinheim, Germany) with a thickness of 20 µm (in its dry state) were sandwiched between two pressure‐sensitive adhesive Tempplate RT select qPCR sealing foils (PSA foil; 100‐µm thick, USA Scientific, Ocala, FL, USA). These PSA foils were patterned by laser cutting (VLS 2.30DT, Universal Laser Systems, Scottsdale, AZ, USA) to form fibroblast seeding areas and microfluidic channels. The bottom layer of the system was fabricated from Flexdym polymer (Eden Tech, Paris, France) – a soft thermoplastic elastomer –^[^ ^42^ ^]^ in a hot‐embossing process. In brief, to fabricate the master mold for the hot‐embossing process, roughly 5–6 mL of SU‐8 3050 photoresist (Micro Resist Technology, Berlin, Germany) were spin‐coated onto a 4‐inch silicon wafer to obtain a 300‐µm thick SU‐8 layer. After curing, the SU‐8‐coated wafer was exposed to UV light through a transparency foil mask featuring the pattern of the microfluidic channels. The exposed SU‐8 wafer was baked, developed, and coated with Trichloro(1H,1H,2H,2H‐perfluorooctyl)silane (Merck) using a chemical vapor deposition method. The microfluidic structure was then transferred to a Flexdym slab using a compact nanoimprint tool (CNI v2.0, NIL Technology, Kongens Lyngby, Denmark). Immediately after the embossing process, the imprinted Flexdym slab was peeled off the master mold, cut into individual units, and stored in an air‐tight box until use. Up to 3 units of the bottom layer can be fabricated in one hot embossing cycle that lasted ≈10 min. Detailed dimensions and layer arrangement of the design are shown in Figure S1 (Supporting Information).

As all structural layers of the system were pre‐cut into defined sizes after their fabrication processes, the assembly of MultiU‐Int microfluidic chip was straightforward. Prior to system assembly, the embossed surface of the Flexdym bottom layer was treated with oxygen plasma (50 W) for 2 min (Harrick Plasma PDC‐002, Harrick Plasma, Ithaca, NY, USA) and sterilized under UV in the laminar flow hood together with PSA foils. CCC was cut into 5 mm × 30 mm strips using sterile dissecting scissors, and the middle layer was assembled by sandwiching three CCC strips between PSA foils to cover individual apical compartments. The middle layer was then bonded onto the top layer using the adhesive that comes with the sticky‐slide 18 well part. Lastly, the Flexdym bottom was aligned with the top and middle layer ensemble so that the microfluidic pattern on Flexdym bottom matched the identical pattern on the PSA foil. All layers were pressed together manually, and the assembled system was kept for at least 1 h at room temperature (RT) in a sterile box to facilitate sealing between the layers. After system assembly, the final height of the apical compartments was 400 µm. A schematic of the fabrication process is shown in Figure S2 (Supporting Information).

To prepare the system chips for experiments, one day before cell seeding, the apical compartments were coated with MaxGel ECM hydrogel solution (Merck) (1:100 dilution in phosphate‐buffered saline (PBS (−/−); without calcium chloride (Ca^2+^) and magnesium chloride (Mg^2+^), Gibco, Thermo Fisher Scientific)) and incubated at 37  °C for 2 h. The coating solution was withdrawn completely from the compartments using a vacuum pump. After being dried overnight under sterile conditions in the laminar‐flow hood, the system was ready to be used for cell‐culture experiments.

### On‐chip Intestinal Epithelial Barrier Model Formation and Chip Operation

The on‐chip IEB model was formed in three main steps: i) establishing a subepithelial stromal niche from day −2 to day 0 of the experiment, ii) IEC loading on day 0, and iii) IEB maturation from day 0 to day 5 of the experiment.

- On day −2 of the IEB formation experiment, the apical compartments of each chip were filled with DMEM medium, supplemented with 20% h.i. FBS, 1× NEAA, and 1× Kanamycin sulfate (IEC expansion medium), and incubated for at least 30 min at 37  °C to rehydrate the CCC. The CCD‐18Co fibroblasts were detached from the cell culture flask using TrypLE Express cell detachment solution (1×; Gibco, Thermo Fisher Scientific). The cell suspension was prepared at a density of 10^5^ cells mL^−1^ in *IEC maintenance medium*. 8 µL of fibroblast suspension were pipetted into each fibroblast seeding area within the basal compartments of the chip. The chip was incubated for 1 h in an incubator at 37  °C and 5% CO_2_ to facilitate fibroblast adhesion to the CCC. Then, 100 µL of *IEC maintenance medium* were added to each basal compartment, while 100 µL of *IEC expansion medium* were added to each of the medium reservoirs for the apical compartments. The chip was kept in an incubator at 37  °C and 5% CO_2_ under static conditions for 2 d. Here, asymmetric concentrations of serum were applied to induce the migration of fibroblasts into the CCC: medium in the apical compartment contained 20% h.i. FBS, while only 10% h.i. FBS was added to the medium in the basal compartments. The fibroblast seeding area also defined the region of interest where all exchanges between the apical and basal sides of the on‐chip IEB happened.
- On day 0 of the IEB formation experiment, after medium exchange, all basal compartments were loaded with cell‐maintenance medium (≈210 µL) and sealed with a PSA foil. Medium was withdrawn from the medium reservoirs of the apical compartments, and the medium that remained within the compartments was exchanged with 30 µL of Caco‐2 and HT29‐MTX E12 cell mixture (9:1 ratio) at a total concentration of 2 × 10^5^ cells cm^−2^ in *IEC expansion medium* (total cell number ≈1.1 × 10^5^ cells in 30 µL). After loading, the chip was closed with a lid, immediately inverted, and incubated for 4 h at 37  °C in the incubator in the inverted position to allow for cell sedimentation and attachment to the CCC. After 4 h, nonadherent cells were flushed from the apical compartments by adding fresh expansion medium to the medium reservoirs only at one side of the chip and placing the chip at an angle. Each reservoir of the apical compartments was filled with 105 µL of *IEC expansion medium*. The sealing of basal compartments was removed, and excess medium was removed from the basal compartments, leaving 105 µL of medium in each compartment. The chips were kept under static conditions overnight in a Nunc 4‐well plate (rectangular well, Thermo Fisher Scientific) – 4 chips per plate, inside a cell‐culture incubator (37  °C, 5% CO_2_).
- On day 1 of the IEB formation experiment, after medium exchange, the plate(s) was placed on a tilting stage (Multi Bio RS‐24, InSphero AG, Schlieren, Switzerland) (Figure S3a, Supporting Information) and tilted by ± 4° with a motion time of 45 s in each direction and a resting time of 58 min in a cell‐culture incubator. On day 3 of the experiment, the medium of the apical compartments was switched to *differentiation medium* – DMEM medium supplemented with 1× NEAA, 1× Kanamycin sulfate, and 1× animal origin‐free Insulin‐Transferrin‐Selenite supplement (Merck). Medium exchanges were performed daily. The on‐chip IEB was ready for further experiments from day 5 on.

### Establishing Co‐cultures of Intestinal Epithelial Barrier Model and Immune Cells

As the four basal compartments of the same on‐chip IEB model can be manipulated separately, at day 5 of the experiment, we inoculated each compartment with 2 × 10^4^ cells of each MNP type or 5 × 10^4^ PBMCs, prepared in 140 µL of *IEC maintenance medium*. For the experiment with MNP subsets, one basal compartment was kept as a no‐MNP control and filled with an equal volume of *IEC maintenance medium*. Meanwhile, PBMCs were added to all four basal compartments. After immune‐cell seeding, the chip was put back on the tilting stage to continue the tilting routine. A full medium exchange was performed in the apical compartments on day 6 following the same procedure described for day 1. At day 6, the medium in the basal compartments of the chip was partially exchanged by gently removing 100 µL of the old medium to avoid removing immune cells and by then adding an equal volume of fresh medium. When a basal dosing of a compound was included, the dosing concentration was adjusted to obtain the desired concentrations on chip.

### Recapitulating Intestinal Inflammation on‐chip

To investigate the exclusive contribution of different immune cell populations in IBD initiation, we kept the external stimuli at a minimum. We dosed the IEB model apically with 10 ng mL^−1^ LPS, derived from *E. coli* bacteria serotype O111:B4 (LPS, Merck), to mimic the presence of Gram‐negative bacteria‐derived endotoxin in the intestinal lumen. To examine LPS uptake by cells of the IEB, FITC‐conjugated LPS (Merck) of the same serotype was used. To trigger inflammation, human recombinant IFN‐γ (Peprotech, Thermo Fisher Scientific) at a final concentration of 20 ng mL^−1^ was added into designated basal compartments of the IEB at day 5 of the experiment. Infliximab (Merck) was dosed basally at a final concentration of 10 µg mL^−1^ at day 6 of the experiment to study the responses of the IEB model‐immune cell co‐cultures to this commonly used therapeutic compound.

### Cell Labeling and Live‐cell Imaging

To monitor IEB model formation on chip, at day 0, IECs were incubated with 1 × 10^−6^ m Calcein AM (excitation/emission (ex/em) 494/517 nm, Thermo Fisher Scientific) in *IEC expansion medium* for 30 min after the nonadherent cells had been removed. Live‐cell imaging was performed on a Nikon TiE inverted microscope (Nikon Europe B.V., Amsterdam, Netherlands) in a wide‐field configuration using a Plan Fluor 4× objective (numerical aperture (NA): 0.13, working distance (WD): 17.1 mm). From day 1 of the experiment on, replenishment of Calcein AM was carried out with the daily medium exchange. 5 × 10^−6^ M BioTracker NucView 405 Blue Caspase‐3 dye (ready‐to‐use stock solution prepared in PBS, ex/em 429/469 nm, Merck) was added to the corresponding medium together with Calcein AM to visualize apoptotic cells. After medium exchange, the on‐chip IEB model was incubated at 37  °C in the cell‐culture incubator for 30–45 min before imaging.

### Permeability Assay

To assess the permeability of the on‐chip IEB model for fluorescent tracers with different sizes, NaFl (376.25 Da, Merck) and 70 kDa RITC‐dextran (Merck) were loaded at a concentration of 5× 10^−6^ m and 200 µg mL^−1^ into the apical compartments, respectively. Normal *IEC maintenance medium* without fluorescent tracers was loaded into the basal compartments. After a 4‐hour static incubation or under the described tilting scheme, 40 µL were sampled from each compartment, and the fluorescence signals of both tracers were measured simultaneously using a microplate reader (Tecan Infinite M1000 Pro, Tecan, Männedorf, Switzerland) (ex/em of NaFl: 495/520 nm, ex/em of RITC‐dextran: 544/576 nm). The apparent paracellular permeability (Papp) was calculated using the following formula

(1) $$\mathrm{Papp}=\frac{V_{basal}*dC_{basal}}{A*dt*C_{apical,t=0}}\to \left(\frac{cm}{s}\right)$$

where *V*_basal_ is the volume of individual basal compartment, *C*_basal_ is the tracer concentration in the basal compartment, *A* is the area of the region of interest, d*t* is the incubation time, and *C*_apical_ is the tracer concentration in the apical compartment.

### Mucus Staining—Alcian Blue‐Periodic Acid Schiff Staining

To detect mucous substances, produced by on‐chip IEB model, the IEB model was first washed with Dulbecco's PBS (PBS (+/+), with calcium chloride (Ca^2+^) and magnesium chloride (Mg^2+^) to remove phenol‐red‐contained medium and then fixed with chilled methacarn fixation solution (60% methanol (Merck) + 30% chloroform (Merck) + 10% glacier acetic acid (Merck)) for 1 h on ice. All reagents used in the next steps were included in the Alcian blue‐periodic acid Schiff (PAS) stain kit from Abcam (Cambridge, UK). After fixation, the Flexdym bottom was removed from the chip, and the fixed IEB model was continuously washed with Milli‐Q water for 5 min before being treated with 3% acetic acid for 2 min at RT. Then, acetic acid was exchanged with Alcian blue (pH 2.5) solution to stain for acidic sulfated mucous substances with an incubation time of 20 min at RT. The stained IEB model was washed for 2 min in running tap water, followed by 2 brief rinses with Milli‐Q water, and covered with a thin layer of water before being imaged using a Leica MZ16 A stereoscope (Leica Microsystem, Wetzlar, Germany). After imaging, water was removed, and the sample was incubated with a periodic acid solution for 5 min at RT. Schiff's solution was applied after a washing step with Milli‐Q water, and the sample was left at RT for 20 min before being washed for 2 min in warm running tap water, followed by 2 brief rinses with Milli‐Q water. Lastly, hematoxylin (modified Mayer's solution) was added and incubated for 2 min to visualize cell nuclei. Washing steps with running water and, afterward, Milli‐Q water were repeated, and the IEB model was imaged again using Leica MZ16 A stereoscope to observe neutral mucins and other substances such as hyaluronic acid and sialomucins.

### Immunofluorescence Staining and High‐resolution Microscopy

The on‐chip IEB model was fixed directly on chip after the experiment. In brief, all culture medium was removed from the reservoirs, then all apical compartments were flushed twice with 200 µL of PBS (+/+) and once with 200 µL of PBS (–/–). All basal compartments were washed twice with 200 µL of PBS (+/+) and once with 200 µL of PBS (‐/‐). The IEB model was fixed with Image‐iT fixative solution (Invitrogen, Thermo Fisher Scientific) for 15 min at RT. The fixed IEB model was rinsed with PBS (–/–) for 5 min on both sides and blocked with BlockAid blocking solution (Invitrogen, Thermo Fisher Scientific) for at least 30 min. Depending on the experiments, different combinations of the following antibodies/stainings were used to stain the on‐chip IEB: Alexa Fluor (AF) 488‐ or 647‐conjugated anti‐ZO‐1 (clone ZO1‐1A12, Invitrogen, Thermo Fisher Scientific) – 1:200 dilution, AF546‐conjugated Ezrin (clone 3C12, Santa Cruz Biotechnology, Dallas, TX, USA) – 1:40 dilution, AF647‐conjugated E‐cadherin (clone EP700Y, Abcam) – 1:100 dilution, AF546‐conjugated mucin‐2 (MUC2, clone H9, Santa Cruz Biotechnology – 1:40 dilution, MaxLight 650‐conjugated polyclonal anti‐alkaline phosphatase, intestinal type (ALPi, USBiological Life Sciences, Salem, MA, USA) – 1:100 dilution, and CellMask green or deep red actin tracking stain (F‐actin, Invitrogen, Thermo Fisher Scientific) – 1:1000 dilution. All antibodies were diluted in BlockAid blocking solution and incubated with the fixed sample overnight at 4 °C. The washing step was repeated, and nuclear counterstaining was performed using NucBlue Live ReadyProbes Reagent (Hoechst 33 342, Invitrogen, Thermo Fisher Scientific) for 40 min at RT. Finally, the IEB model was washed and stored in PBS (−/−) at 4 °C for a maximum of a week until image acquisition. Before imaging, PBS was removed from all compartments, and the Flexdym bottom was removed from the chip. A microscopic cover glass with 1.5H thickness (24 mm × 60 mm, 170‐µm thick cover glass, Marienfeld GmbH, Lauda‐Königshofen, Germany) was mounted to the bottom of the chip using ProLong glass antifade mounting medium (Invitrogen, Thermo Fisher Scientific). All fibroblast seeding areas on the basal sides of IEB were covered with 8 µL of ProLong glass antifade mounting medium. Fluorescence images and Z‐stacks of the IEB model were acquired with a Nikon X‐Light v3 inverted spinning‐disk confocal microscope (Nikon Europe B.V.) using a Plan Apo λ 10× (NA: 0.45, WD: 4 mm), Plan Apo λS 25× (NA: 1.05, WD: 0.55 mm), or Plan Fluor 40× objective (NA: 1.3, WD: 0.24 mm).

Indirect IF staining was used to detect ECM proteins, produced by fibroblasts, such as laminin‐α5 and collagen IV. Sample fixation was performed as described for the IEB sample preparation, and the sample was incubated overnight at 4  °C with unconjugated primary antibodies: rabbit polyclonal anti‐human laminin α5 (Bioss Antibodies, Woburn, MA, USA) – 1:200 dilution and goat polyclonal anti‐human collagen IV (SouthernBiotech, Birmingham, AL, USA) – dilution 1:200, in 0.1% bovine serum albumin (BSA, Merck) in PBS (‐/‐). Next, the sample was washed with PBS (‐/‐) and incubated for 90 min at RT with fluorophore‐conjugated secondary antibodies: AF488‐conjugated donkey anti‐rabbit IgG H&L, pre‐adsorbed (Abcam) – 1:200 dilution, and AF647‐conjugated donkey anti‐goat IgG H&L, pre‐adsorbed (Abcam) – 1:200 dilution. The washing step was repeated, and the sample was stored in PBS (‐/‐) at 4 °C until image acquisition. Sample mounting and image acquisition were carried out as described for the IEB sample.

### Second Harmonic Generation Microscopy

We visualized and assessed possible changes in CCC under different experimental conditions by SHG microscopy using an inverted Zeiss LSM 980 multiphoton microscope (Carl Zeiss AG, Oberkochen, Germany) and a C‐Apochromat 40× objective (NA: 1.1, WD: 0.62 mm). As the SHG effect allows for label‐free visualization of the collagen network, multiplexed imaging was conducted to visualize CCC and IF staining. The two‐photon laser at 820 nm was used for SHG, and emission was detected at 410±10 nm with a multialkali‐photomultiplier module detector.

### Multiplex Enzyme‐linked Immunosorbent Assay

After collecting the cell culture supernatant into a low‐binding Nunc 96‐well polypropylene storage microplate (Thermo Fisher Scientific), we centrifuged the plate at 500 × g for 10 min to remove cell debris. The supernatant was then transferred to a new storage plate of the same type, and BSA was added to reach a final concentration of 0.1%. The supernatant was stored at ‐20 °C until use. We employed customized bead‐based multiplex assays according to the manufacturer's protocol (BioRad, Hercules, CA, USA and Biolegend) to measure IL‐8, IP‐10, MCP‐1, TNF α, IL‐1β, IL‐6, IL‐12p40, and GM‐CSF inside the supernatant. Bio‐Plex MAGPIX multiplex reader (Biorad) or BD LSRFortessa flow cytometer (BD Bioscience, Franklin Lakes, NJ, USA) with a high‐throughput sampler was used to acquire data from the multiplex assay. Fibroblast‐derived collagenase – MMP‐1 – was quantitated using a human MMP‐1 enzyme‐linked immunosorbent assay kit (Abcam) and a microplate reader (Tecan Infinite M1000 Pro, Tecan).

### Flow Cytometry

Surface marker expressions in MNs, MF, iDCs, and PBMCs were analyzed by flow cytometry before the cells were loaded into the MultiU‐Int chips (day 5), and 2 d after on‐chip co‐culturing (day 7). At day 7, as a portion of MNPs were expected to adhere to or have penetrated the CCC during the experiment, MNPs of the same type and treated with the same stimuli during the experiment were pooled to ensure sufficient cell numbers for flow cytometry analyses. Adherent MFs were detached from the cell culture well plate using cold 2 × 10^−3^ M ethylenediaminetetraacetic acid (EDTA) (Invitrogen, Thermo Fisher Scientific). In brief, the cells were washed twice with PBS (−/−) and then incubated with cold EDTA on ice for 15 min. Nonadherent MNs, iDCs, and PBMCs were collected directly from the cell‐culture well plate for analysis. Cells were washed twice with flow cytometry buffer (5% FBS in PBS (−/−)), and the cells were collected by centrifugation at 4  °C for 5 min at 300 × *g*. To distinguish viable cells from dead cells, cells were first stained with fixable viability stain 575V (FVS575V, BD Biosciences) for 15 min at RT. After being washed twice with cytometry buffer, cells were stained with fluorophore‐conjugated monoclonal antibodies against specific sets of surface markers as follows: i) MN: PE conjugated CD14 (clone 63D3), APC/Cy7‐conjugated CD16 (clone 3G8), PE/Cy7‐conjugated CD209 (clone Ber‐ACT8), APC‐conjugated CD64 (clone 10.1), and BV421‐conjugated CD163 (clone GHI/61); ii) MF: PE/Cy7‐conjugated CD11b (clone ICRF44), PE‐conjugated CD14, APC‐conjugated CD64, and BV421‐conjugated CD163; iii) iDC: BV421‐conjugated CD11c (clone 3.9), PE‐conjugated CD14, APC‐conjugated CD64, and PE/Cy7‐conjugated CD209, and iv) PBMCs: BV711‐conjugated CD4 (clone L200), PE‐conjugated CD14, APC‐conjugated CD64, PE/Cy7‐conjugated CD209, and BV421‐conjugated CD163. All antibodies were purchased from BioLegend (San Diego, CA, USA), except for APC‐conjugated CD64 and BV711‐conjugated CD4, which was purchased from BD Biosciences. Additionally, THP‐1 NF‐κB‐eGFP cells, the NF‐κB pathway of which was activated, were GFP‐positive. Upon labeling, cells were washed, suspended in cytometry buffer, and analyzed with a BD LSR Fortessa flow cytometer (BD Biosciences). 10 000 events were recorded for each sample. For compensation, single‐color controls for antibodies were prepared using OneComp eBeads compensation beads (Invitrogen, Thermo Fisher Scientific) using the same amount of antibody for one test with cells. Compensation control for FVS575V was prepared with ArC Amine Reactive Compensation Bead Kit (Invitrogen, Thermo Fisher Scientific). For PBMC staining, Human TruStain FcX (Fc Receptor Blocking Solution, Biolegend) was added to prevent nonspecific binding. Except for forward scatter (FCS) and side scatter (SSC) settings, the same fluorescence detector (PMT) voltage setting was used to acquire data for the compensation controls and cell samples. For on‐chip experiments, the same panels and analyzing parameters of the analysis at day 5 were used to analyze cells collected from the on‐chip culture to reveal changes in marker expression. Unstained controls were included in all analyses to support gating. We used the FlowJo software (BD Biosciences) to perform compensation, data analysis, and data visualization. Generally, FCS and SSC gating was applied to exclude debris, and then FCS‐A and FCS‐H gating was used to remove doublets from further analyses. We used a single‐parameter histogram to examine the expression of the markers of interest, and two‐parameter dot plots were included to examine the co‐expression pattern of a pair of markers.

### Flow Simulation

Our MultiU‐Int microfluidic chip relies on a pumpless‐, gravity‐based perfusion system to generate a bidirectional flow within the apical compartments of the chip. The average fluid flow rate and shear stress on the cell surface within the compartments were estimated based on i) the induced pressure difference between the two fluidic reservoirs at the two ends of each apical compartment when the chip was periodically tilted at a defined angle, and ii) the geometry‐dependent hydraulic resistance of the compartment. A parameter sweep of tilting angles from 3° to 10° with an increment of 1° was conducted using the computational fluid dynamics module in COMSOL Multiphysics software (COMSOL, Inc., Stockholm, Sweden). The chosen tilting angle of 4° yielded an average shear stress of 0.025 Pa, which falls into the physiologically relevant regime of 0.0002‐0.08 Pa for intestinal cells.^[^ ^103^ ^]^

### Data Analysis

Microscope images were processed and analyzed by using the Nikon NIS‐Elements Advanced Research (Nikon Europe B.V.), ZEN 3.3 (blue edition, Carl Zeiss AG), Imaris (Oxford Instruments, Oxford, UK), or ImageJ software. We used the Bio‐Plex Manager software (BioRad), LEGENDplex data analysis software Suite (Biolegend), Microsoft Excel (Microsoft, Redmond, WA, USA), and GraphPad Prism 7 software (GraphPad Software, San Diego, CA, USA) to analyze data obtained from the multiplexed assay. This data was statistically analyzed with one‐way or two‐way ANOVA, depending on the data set, and visualized using GraphPad Prism 7 software. All statistical results were represented as mean ± standard deviation (SD) with a significance of P < 0.05 unless indicated differently (ns: not significant, *: *p* < 0.05, **: *p* < 0.01, ***: *p* < 0.001, and ****: *p* < 0.0001). Finally, all data was organized for visualization by Adobe Illustrator (Adobe Inc., San Jose, CA, USA) using input files from the aforementioned software.

## Conflict of Interest

The authors declare no conflict of interest.

## Author Contributions

O.T.P.N conceptualized the project, designed and fabricated the microfluidic chip, designed and planned experiments, performed all experiments, analyzed and interpreted data, and wrote the manuscript. P.M.M conceptualized the project and the design of the microfluidic device, was involved in scientific discussions during the first stage of the project and provided feedback for the manuscript. A.H. coordinated the project, was involved in the scientific consideration, and edited the manuscript. C.L. conceptualized the project, oversaw the experimental plans, interpreted data, and edited the manuscript. All authors contributed to writing the manuscript and approved the submitted version.

## Supporting information

Supporting Information

Supplemental Video 1

Supplemental Video 2

Supplemental Video 3

## Data Availability

The data that support the findings of this study are available from the corresponding author upon reasonable request.
