# Supplementary material for: A Versatile Intestine‐on‐Chip System for Deciphering the Immunopathogenesis of Inflammatory Bowel Disease
Source: Adv Healthc Mater. 2024 Feb 11;13(7):2302454. doi: 10.1002/adhm.202302454 (PMC11468350; doi:10.1002/adhm.202302454)
Supplement: Supplementary file 1 — Supporting Information [file ADHM-13-2302454-s002.pdf]

ADVANCED HEALTHCARE MATERIALS

# Supporting Information

for *Adv. Healthcare Mater.*, DOI 10.1002/adhm.202302454

A Versatile Intestine-on-Chip System for Deciphering the Immunopathogenesis of Inflammatory Bowel Disease

*Oanh T. P. Nguyen*, Patrick M. Misun, Andreas Hierlemann* and Christian Lohasz*

# Supporting Information

**A Versatile Intestine-on-Chip System for Deciphering the Immunopathogenesis of Inflammatory Bowel Disease**

*Oanh T.P. Nguyen*, Patrick M. Misun, Andreas Hierlemann*, and Christian Lohasz*

## Supplementary materials and methods

### *1. Generation of different types of mononuclear phagocytes*

We used the human monocytic cell line THP-1, transfected with an NF-κB-eGFP reporter construct, as a monocyte (MN) model, which was further differentiated into MN-derived macrophages (MFs) and immature dendritic cells (iDCs). TLR4-dependent activation of the NF-κB pathway in THP-1-derived MNs and MFs has been well characterized in many studies.[116]–[119] As shown in Figure S7a (i), THP-1 cells grew in suspension and featured the typical round morphology of MNs in an *in-vitro* culture. Upon stimulation with LPS, THP-1 cells showed a dose-dependent expression of eGFP-tagged NF-κB TF, suggesting LPS recognition and induction of downstream signaling cascades in these cells (Figure S7a, (ii)). For the differentiation of THP-1 MNs into MFs, cells were incubated for 48 hours with a low concentration of phorbol 12-myristate-13-acetate (PMA, 5 nM). The PMA concentration was selected to be lower than that used in other studies (6 - 500 nM) to avoid undesired activation of obtained MFs. The cells cultured under these conditions featured adherent and spreading morphology, which is characteristic of MFs (Figure S7b, (i)). Successful differentiation of THP-1 MN into MFs was further confirmed by fluorescence microscopy, as the differentiated cells expressed a high level of NF-κB-eGFP, which is known to be upregulated in PMA-induced MFs (Figure S7b, (ii)).[120], [121] To obtain THP-1-derived iDCs, we treated THP 1 MNs with IL-4 and GM-CSF for 5 days[115] with periodic renewal of the cell-culture medium. As shown in Figure S7c (i), on day 5, THP-1-derived iDCs remained in suspension and displayed the characteristics of MN-derived iDCs, such as a mildly ruffled cell surface and the formation of dendrites.[122], [123] The obtained iDCs were negative or expressed very low levels of eGFP-tagged NF-κB (Figure S7c, (ii)). Finally, we used flow cytometry to assess cell size, complexity, and expression of the surface marker CD14. CD14 is one of the main differentiation markers expressed on the surface of myeloid lineage cells, such as MN, MF, and iDC. It is also considered a germline-encoded pattern-recognition receptor (PRR)

that enhances the detection of LPS by the TLR4-associated LPS receptor complex.[124]

When compared to THP-1 MNs, THP-1-derived MFs and iDCs showed an increased cell size (as shown by forward scatter; FCS; Figure S7d, (i)) and increased CD14 expression (Figure S7d, (ii)). Additionally, THP-1-derived iDCs exhibited higher granularity than MNs (as shown by the side scatter; SSC; Figure S7d, i). Overall, the three THP-1-derived cell types differed from each other in terms of morphology, expression of NF-κB and CD14, cell size, and relative cell complexity.

## *2. Immune responses by mononuclear phagocytes on-chip*

### *2.1. Monocyte*

Cell surface markers, NF-κB TF expression, and cell viability of MNs were analyzed at day 5 and day 7 post-IEC seeding and are summarized in Figure S8a (i). An example of the gating strategies used to analyze all markers is shown in Figure S11a. Before on-chip co-culturing, less than 10% of THP-1-derived MNs expressed the classic MN cell-surface marker CD14,[123], [124] which was in agreement with previous studies on CD14 expression in THP-1.[127] THP-1-derived MNs expressed negligible levels of CD16, a non-classical MN cell surface marker. The undifferentiated state of these cells was confirmed by the absence of NF-κB TF expression and very low expression levels of the MF cell-surface markers CD64 (9%) and CD163 (2%). As shown in Figure S11b (ii), only a few cells were double-positive for CD14 and CD64. Most THP-1-derived MNs also expressed the DC-specific CD209 surface marker (DC-specific-intracellular adhesion molecule 3-grabbing non-integrin (DC-SIGN)). Indeed, CD209 expression by THP-1-derived MNs has been reported in other work, albeit in a lower proportion.[128]

After 2 days in an on-chip co-culture with an IEB model without a basal inflammatory stimulus, we observed an increase in CD14$^{+}$ (from 8% to 33-35%) and CD64$^{+}$ (from 9% to 47-54%) populations, regardless of the apical LPS content (Figure S8a, (i)). While IFN-γ treatment did not affect the CD14$^{+}$ population, it significantly enhanced the CD16$^{+}$ and CD64$^{+}$ populations (Figure S8a) to approximately 12% and 91% of the single-cell population, indicating a shift to an inflammatory phenotype.[81], [82] Upon additional Infliximab treatment, the CD64$^{+}$ population remained constant, whereas the level of CD14$^{+}$ cells decreased by 10%. A similar decrease in CD14 expression caused by Infliximab therapy has been (i) associated with decreased MN activation in UC patients and (ii) considered one of the early markers for a positive therapeutic response.[84]

Chemo/cytokine analysis of the supernatant of the IEB model-MN co-cultures revealed that inflammatory stimuli caused a slight increase in the levels of IEC-derived IL-8, while IP-10 levels remained low and unchanged (Figure S8b). The application of LPS alone did not alter the MN-derived chemo/cytokine profiles within the basal compartment of the IEB (Figure S8c). When IFN-$\gamma$ was applied to the basal side of the IEB model, TNF-$\alpha$ and IL-1$\beta$ levels increased drastically, indicating the existence of an inflammatory milieu. In agreement with its effect on CD14 expression, Infliximab administration effectively neutralized TNF-$\alpha$ secretion by MNs and maintained the secretion of TNF-$\alpha$ at an unstimulated level. IEC-derived IL-8, on the other hand, was stably produced at a high level regardless of Infliximab administration.

### *2.2. Macrophage*

Cell surface markers, NF-$\kappa$B TF expression, and cell viability of MFs were analyzed at day 5 and day 7 post IEC seeding (Figure S9a) using the same gating strategy as shown in Figure S8a. Flow cytometry analysis of the THP-1-derived MFs confirmed the successful differentiation of THP-1 MNs into MFs, as approximately 70% of the obtained MFs were double-positive for the eGFP-tagged NF-$\kappa$B and MF-specific marker CD11b (Figure S12b). This result further supports the microscopic observations of MF-specific morphology and PMA-induced NF-$\kappa$B TF expression, as shown in Figure S6b. The obtained MFs assumed a non-activated state (M0-like MF) as shown by low CD14 expression and rare expression of differentiation markers, such as CD64 and CD163.

At day 7 post IEC seeding, after 2 days of on-chip co-culturing, CD14$^+$ and CD64$^+$ populations significantly increased in an LPS-independent manner (Figure S9a), indicating MF maturation. When the basal compartments of the on-chip co-culture were exposed to IFN-$\gamma$, the CD64$^+$ population became dominant, accounting for 83% of the single-cell population. This drastic increase in the CD64$^+$ population indicated that the M0-like MFs polarized into a pro-inflammatory M1-like phenotype.[83] Only 10% of the single-cell population acquired an M2-like MF phenotype with CD163 expression. Infliximab treatment slightly enhanced the NF-$\kappa$B$^+$, CD14$^+$, and CD64$^+$ populations but did not change the expression pattern of CD11b and CD163 markers. More details of the changes in the co-expression of CD11b and NF-$\kappa$B are shown in Figure S12c-S12d.

In general, the co-culture of the IEB model with MFs led to a higher production of IEC-derived IL-8 and IP-10 as compared to co-cultures with MNs **(**Figure S9b**)**. However, unlike what was observed in the IEB model-MN co-cultures, the levels of IEC-derived IL-8

and IP-10 decreased after IFN-γ was administered to the basal compartments of the IEB model. IL-8 and IP-10 levels remained low after Infliximab administration. Quantification of MF-derived cytokines showed that apical dosing with LPS alone increased the production of GM-SCF significantly (Figure S9c).[129] When LPS and IFN-γ were applied simultaneously to the apical and basal sides, the basal release of all chemokines/cytokines diverged. While the level of IL-6 remained unchanged, the levels of other MF-derived cytokines decreased, especially those of IL-1β and GM-CSF. Infliximab did not significantly alter the levels of TNF-α, GM-CSF, and IL-1β. We hypothesized that (i) high levels of GM-CSF, released upon LPS stimulation, and (ii) the emergence of anti-inflammatory M2 MF may be responsible for the decreased expression of pro-inflammatory cytokines. Although GM-CSF has been commonly considered as pro-inflammatory cytokine, recent studies have shown that in IBD, it can downregulate inflammatory responses as a part of a complex signaling cascade to restrain IBD pathogenesis.[86]–[89]

### *2.3. Immature dendritic cell*

Similar to MNs and MFs, cell surface markers, NF-κB TF expression, and cell viability of iDCs were analyzed by flow cytometry at day 5 and day 7 post IEC seeding using the gating strategy shown in Figure S8a. Marker expression of iDCs on day 5 showed that IL-4- and GM-CSF treatment induced the expression of the MN cell-surface marker CD14 (75%) and the DC-specific cell-surface marker CD209 (97%) (Figure S10a). More iDCs expressed CD14 and CD209 in comparison to THP-1-derived MNs; in particular, the fraction of CD14-expressing cells increased nine-fold (75% versus 8%). The expression of the MN marker CD14 together with the low expression of the CD11c marker indicated that the obtained DCs acquired an immature phenotype.[130], [131] In agreement with microscopic observation of NF-κB TF expression (Figure S6c), only a small fraction of iDCs (6%) expressed NF-κB, evidenced by cytometry analysis.

After 2 days in on-chip co-cultures with the IEB model, the $CD11c^+$ population increased by 13-16%, even in the absence of LPS, indicating the maturation of iDCs to a more mature phenotype. The presence of LPS in the apical compartment significantly increased the $CD64^+$ iDC population (from 13% to 74%). A plausible explanation for their maturation is their capacity to directly sample the apical content for early detection of antigens (i.e., bacterial endotoxins),[79] and their detection of the LPS that was present at the apical side of the IEB model.[80] During LPS-induced maturation, iDCs also upregulated MN- and MF-specific markers (i.e., CD14 and CD64) as reported previously.[103] IFN-γ treatment further increased

NF-κB$^+$, CD209$^+$, CD11c$^+$, and CD64$^+$ populations, especially the latter two populations (Figure S10a and Figure S13), which indicates that more iDCs matured and were activated under LPS- and IFN-γ-dependent inflammatory conditions. Upon Infliximab treatment, iDCs maintained their activated state, as indicated by the high percentages of NF-κB$^+$ (40%), CD11c$^+$ (42%), and CD64$^+$ (90%) cells (Figure S10a).

Unlike MFs, the central roles of iDCs, or DCs in general, in the subepithelial stromal niche of the IEB include to sample apical antigens and to subsequently initiate inflammatory responses of T cells via antigen presentation.[132] Therefore, we expected that the direct inflammatory response of iDCs would be less pronounced than those of MNs and MFs. As shown in Figure S10b, apical dosing with LPS did not alter the levels of IEC-derived and iDC-derived chemo/cytokines. Simultaneous application of LPS and IFN-γ on the apical and basal sides induced the upregulation of GM-CSF only (Figure S10c). Upregulation of GM-CSF production by CD11$^+$ DCs in response to the invasion of pathogenic bacteria has been reported in animal models,[85] suggesting a proper inflammatory response by DCs in our system. In the future, the inclusion of relevant T cells in on-chip IEB model-iDCs co-cultures will help to reveal more details on the role of iDCs in IBD pathogenesis. As TNF-α takes part in driving DC activation,[133] the blocking of TNF-α by Infliximab led to decreased TNF-α-induced DC activation, indicated by decreased levels of GM-CSF and TNF-α levels in the basal compartments of the on-chip co-cultures.

We obtained high viability for all immune cells in our on-chip co-cultures (Figure S8a, S9a, and S10a). Among the three cell types, iDCs showed the highest sensitivity to stimuli, as their viability fluctuated. While their viability remained over 70% under all non-inflammatory conditions, a drop of 19-37% was observed upon treatment with IFN-γ for 2 days. This increased cell death is most likely related to DC activation, which induced multiple pathways that subsequently led to programmed cell death in DCs.[134]

**Supplementary tables**

**Table S1.** Average MMP-1 concentration detected in the basal compartments of on-chip co-cultures at Day 7 (shown as mean ± standard deviation (ng/mL))

| | **- LPS** | **+ LPS** | **+ LPS, + IFN-γ** | **+ LPS, + IFN-γ, + Infliximab** |
|---|---|---|---|---|
| **No MNPs** | 48 ± 9 | 58 ± 6 | 45 ± 7 | 43 ± 6 |
| **+ MN** | 52 ± 14 | 50 ± 2 | 41 ± 6 | 36 ± 3 |
| **+ MF** | 59 ± 4 | 62 ± 4 | 60 ± 14 | 53 ± 6 |
| **+ iDC** | 58 ± 4 | 64 ± 3 | 59 ± 10 | 65 ± 3 |

## Supplementary figures

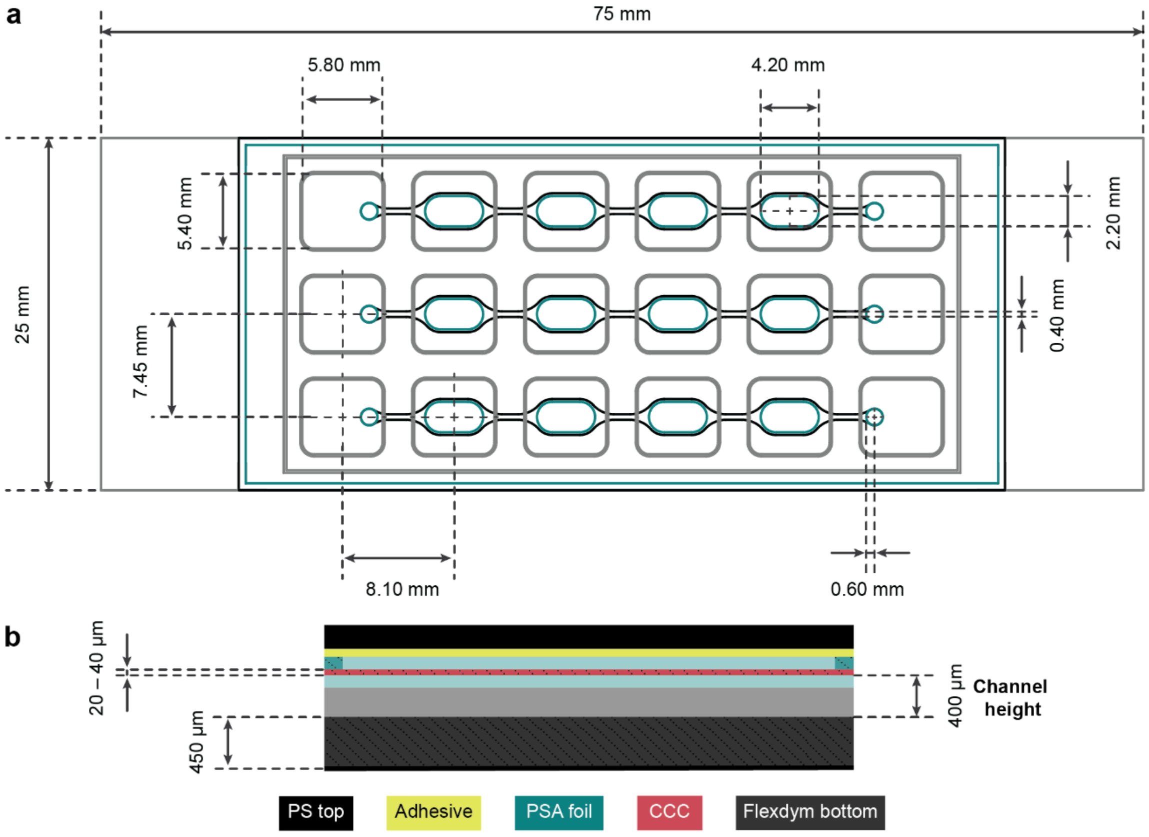


**Figure S1.** a) Detailed dimensions of the MultiU-Int microfluidic chip. b) Assembly of all layers after bonding.

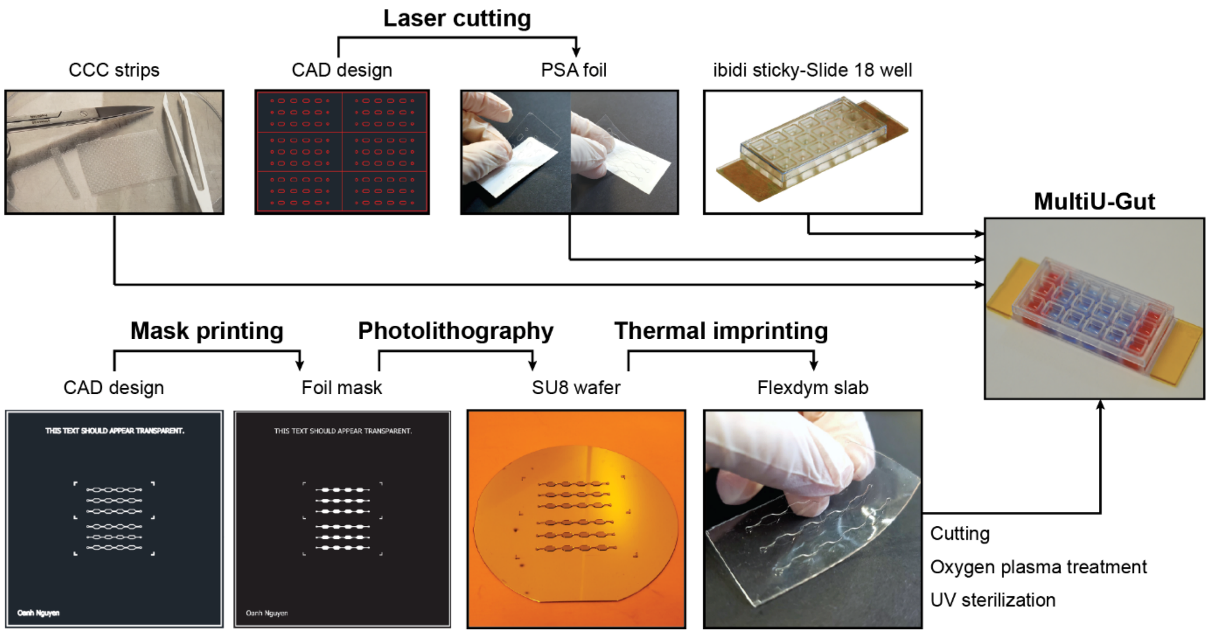


**Figure S2.** Overview of the workflow of the MultiU-Int microfluidic chip fabrication

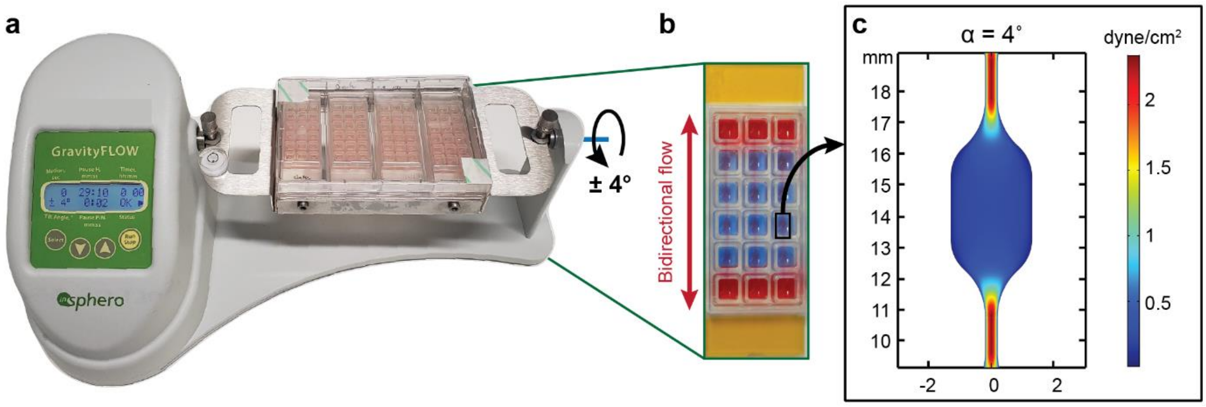


**Figure S3.** a) Arrangement of the MultiU-Int microfluidic chips on a GravityFlow™ tilting device. As an example, two well plates – each contained four microfluidic chips – were stacked on top of each other for experiment parallelization. The whole setup was kept in a cell-culture incubator at 37°C, 5% $CO_2$, and 98% humidity. b) The direction of the flow within the apical compartments of the microfluidic chip. c) A simulation of the average shear stress at the surface of an on-chip IEB model. The average shear stress amounted to 0.025 Pa in each individual apical region.

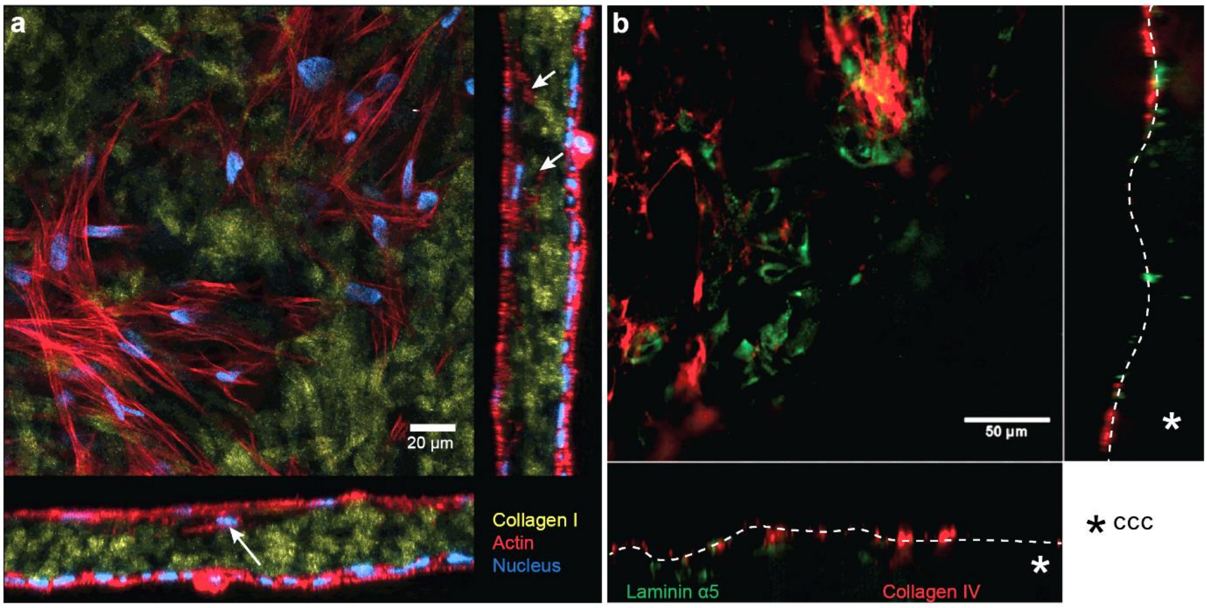


**Figure S4.** a) Migration of fibroblasts into the CCC (arrows) as evidenced by SHG microscopy and IF staining. Scale bar: 20 µm. b) *De novo* deposition of ECM proteins, such as Laminin α5 and Collagen IV, by fibroblasts that migrated into and resided within the CCC prior to IEC seeding. Scale bar: 50 µm. White dashed line: Upper border of the CCC. White asterisk: CCC.

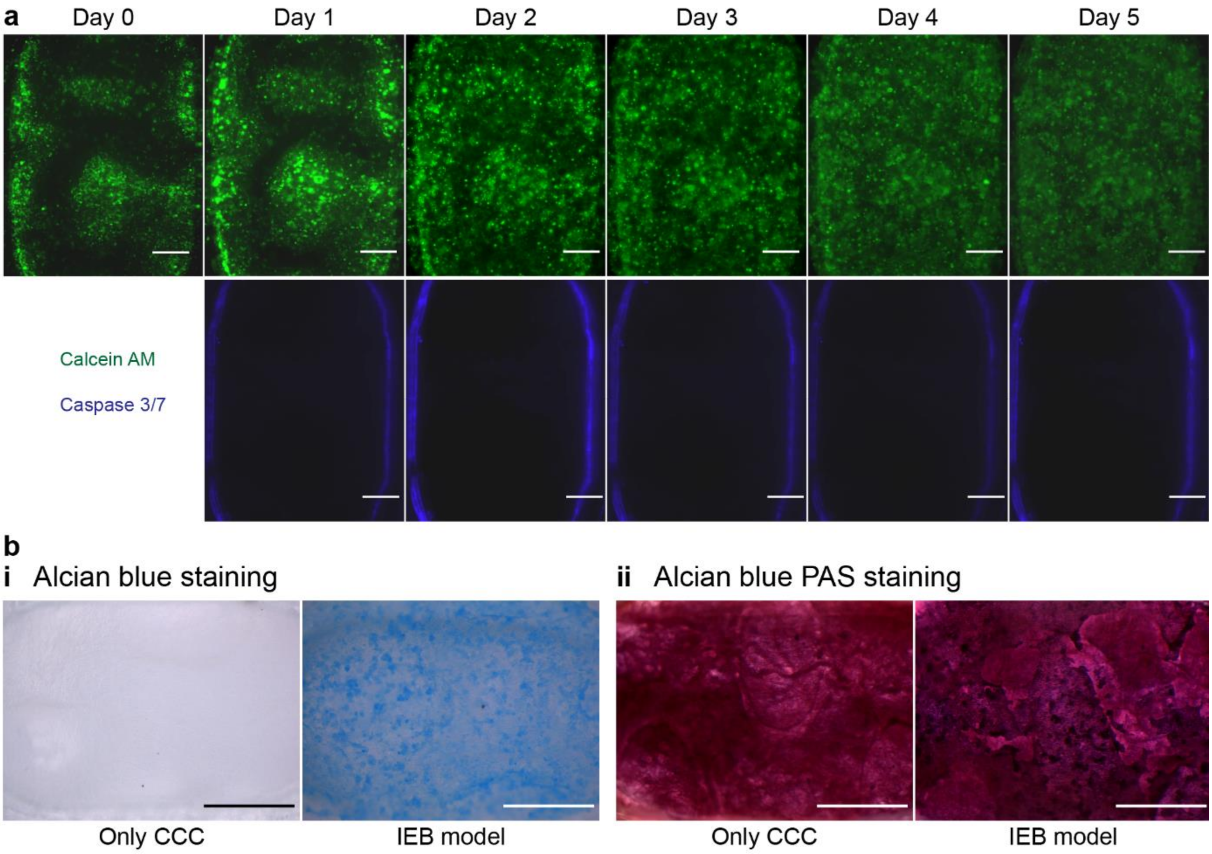


**Figure S5.** a) On-chip cell viability during IEB model formation. Calcein AM Green staining showed good cell viability and coverage during 5 days of an on-chip IEB model formation. Scale bar: 500 μm. b) Characterization of the mucus layer produced by on-chip IEB models: (i) Alcian blue staining for acid mucins (blue: acid mucins), and (ii) Alcian blue PAS staining for other mucins (dark blue: nuclei, magenta: neutral mucins). Scale bars: 1 mm.

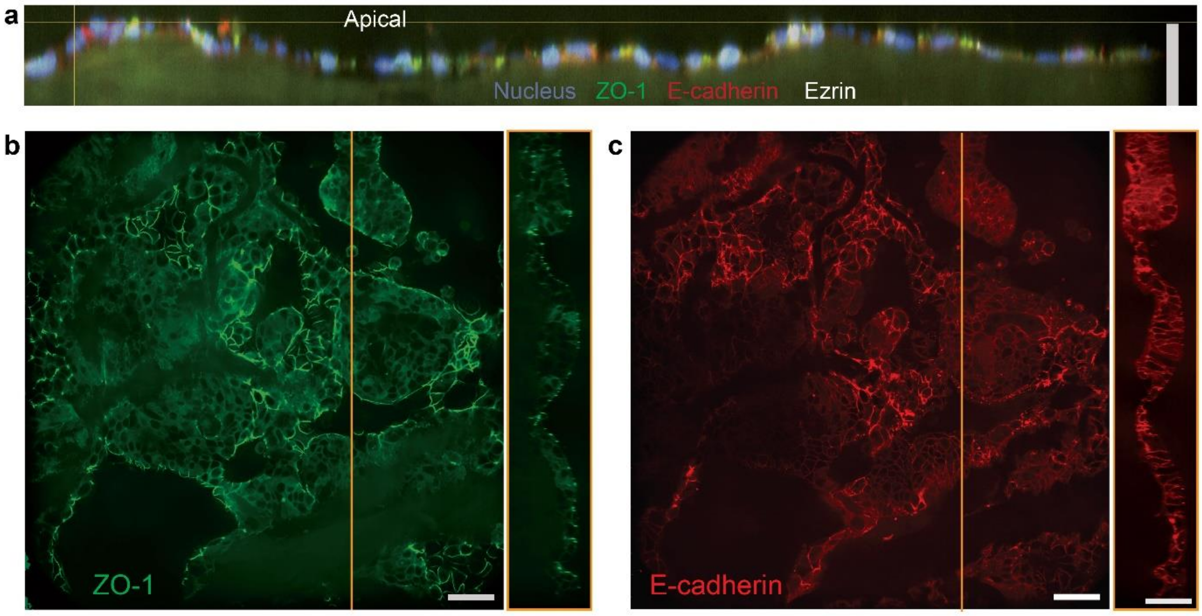


**Figure S6.** a) A z-cross section of a polarized IEB model – formed under static culture conditions for 5 days. Polarization of the on-chip IEB formed under dynamic culture conditions as shown by immunofluorescence staining of b) apically localized tight junction protein ZO-1 and c) basolaterally localized E-cadherin. The panel on the left of each subfigure shows one single z-plane and the panel on the right shows the yz-section in single color of the same z-stack featured in Figure 3c (i). Scale bars: 50 µm.

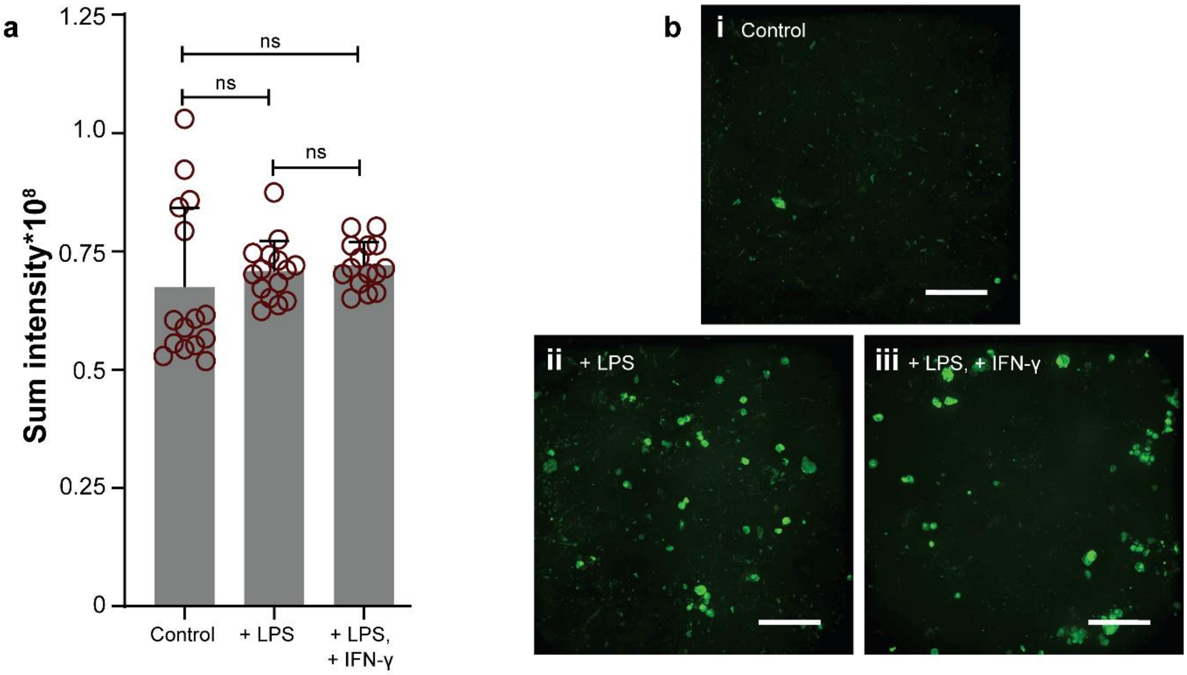


**Figure S7.** a) Relative changes in TLR4 level expressed on the surface of IECs (n = regions of interest), and b) LC3 autophagy marker, expressed by IECs when the on-chip IEB models were exposed to different inflammatory stimuli (LPS, IFN-γ). Scale bars: 100 µm. (ns: not significant).

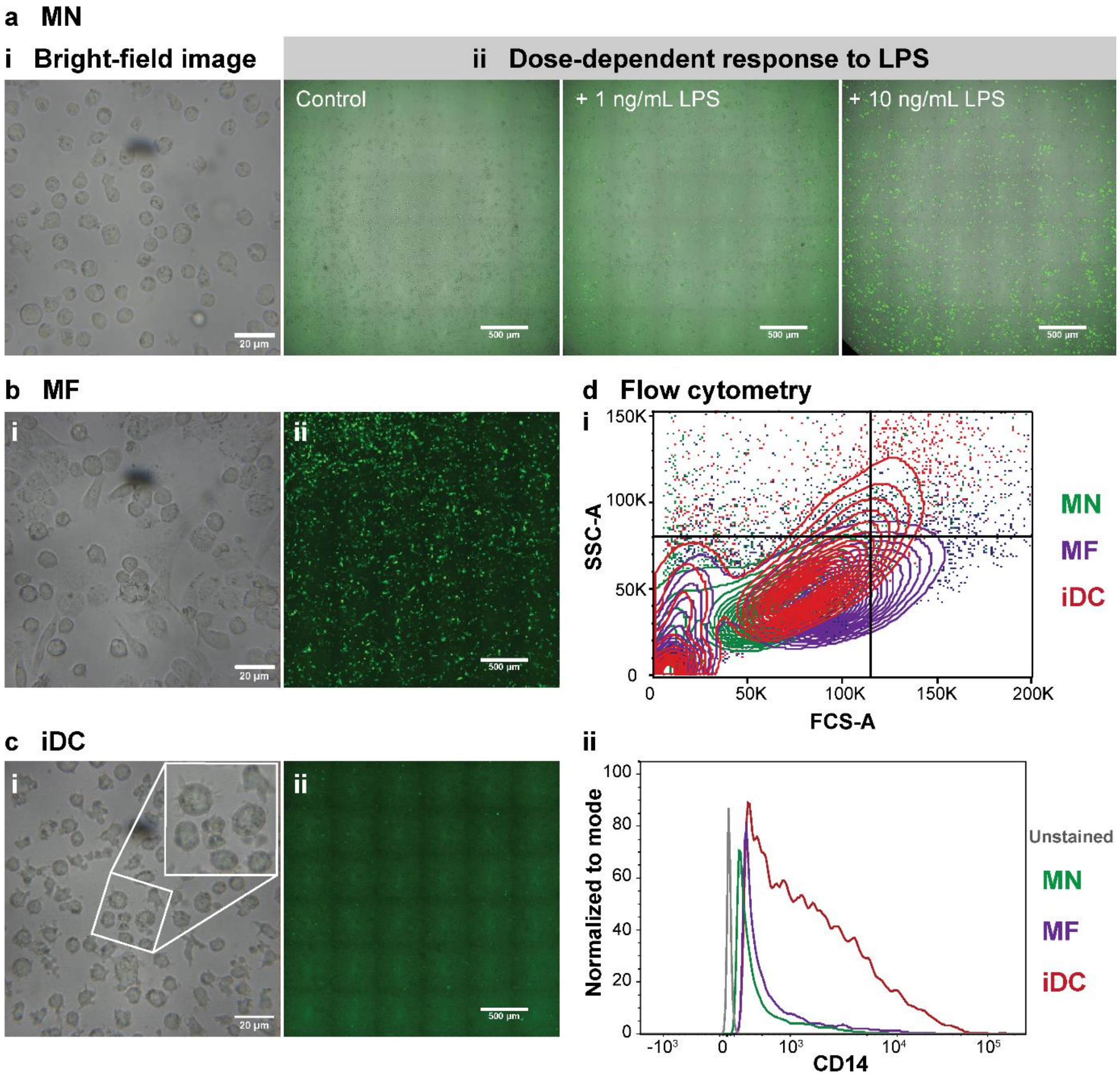


**Figure S8.** Characterizations of mononuclear phagocytes used in this work. a) (i)Morphology of THP-1 NF-κB eGFP-derived MNs as shown by bright-field images. Scale bar: 20 µm. (ii) THP-1 NF-κB eGFP-derived-MNs showed proper dose-dependent upregulation of NF-κB expression by LPS. The cells featured an adherent phenotype, similar to that of MFs, after 48 hours of treatment. Scale bars: 500 µm. b) Differentiation of MNs to MFs by PMA treatment: (i) Morphology of MN-derived MFs 48 hours after PMA exposure. ii) Upregulated expression of NF-κB TF in MFs after 48 hours of PMA treatment (left), indicated by a high expression of the eGFP reporter. c) Differentiation of MNs to iDCs by IL-4 and GM-CSF cytokines: (i) Morphology of MN-derived iDCs at day 5 of cytokine treatment. Scale bar: 20 µm. (ii) In contrast to MFs, iDCs expressed a very low level of the eGFP reporter, indicating a low expression of NF-κB TF. Scale bars: 500 µm. d) Flow-cytometry analysis of the obtained cell types: (i) Forward-scatter (FSC) versus side-scatter gating (SSC) showed that MFs and iDCs had increased cell sizes as compared to MNs. Additionally, iDCs acquired the highest granularity among the three cell types. The MN population was contained in the lower left quadrant. (ii) Histogram showed a shift of CD14 expression of the three cell types.

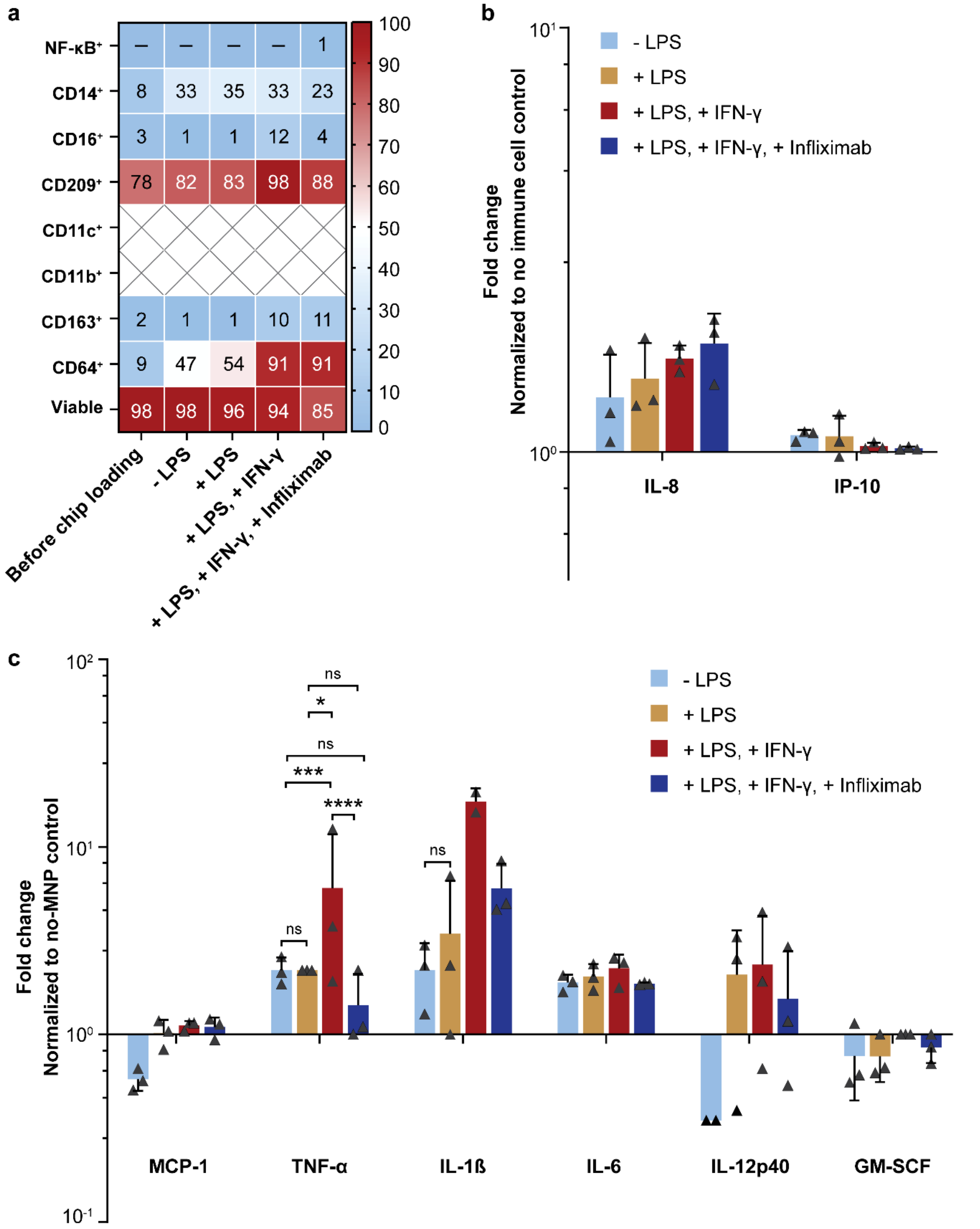


**Figure S9.** a) Heat map representation of flow cytometry analysis results of the marker expression of MNs before and after 2 days in co-culture with the IEB model upon applying different stimuli. The fraction of positive cells (%) is shown in each box of the heat map. b) IEC-derived chemo/cytokine profiles in on-chip co-cultures with MNs at day 7 (n = 3). Data is represented as x-fold change with respect to the baseline of the "no-MNP" control. c) Full MN-derived cytokine profile of on-chip co-cultures of IEB models with MNs at day 7 (n = 3). Data is represented as x-fold change with respect to the baseline of the "no-MNP" control (ns: not significant, *$p < 0.05$, **$p < 0.01$, ***$p < 0.001$, and ****$p < 0.0001$).

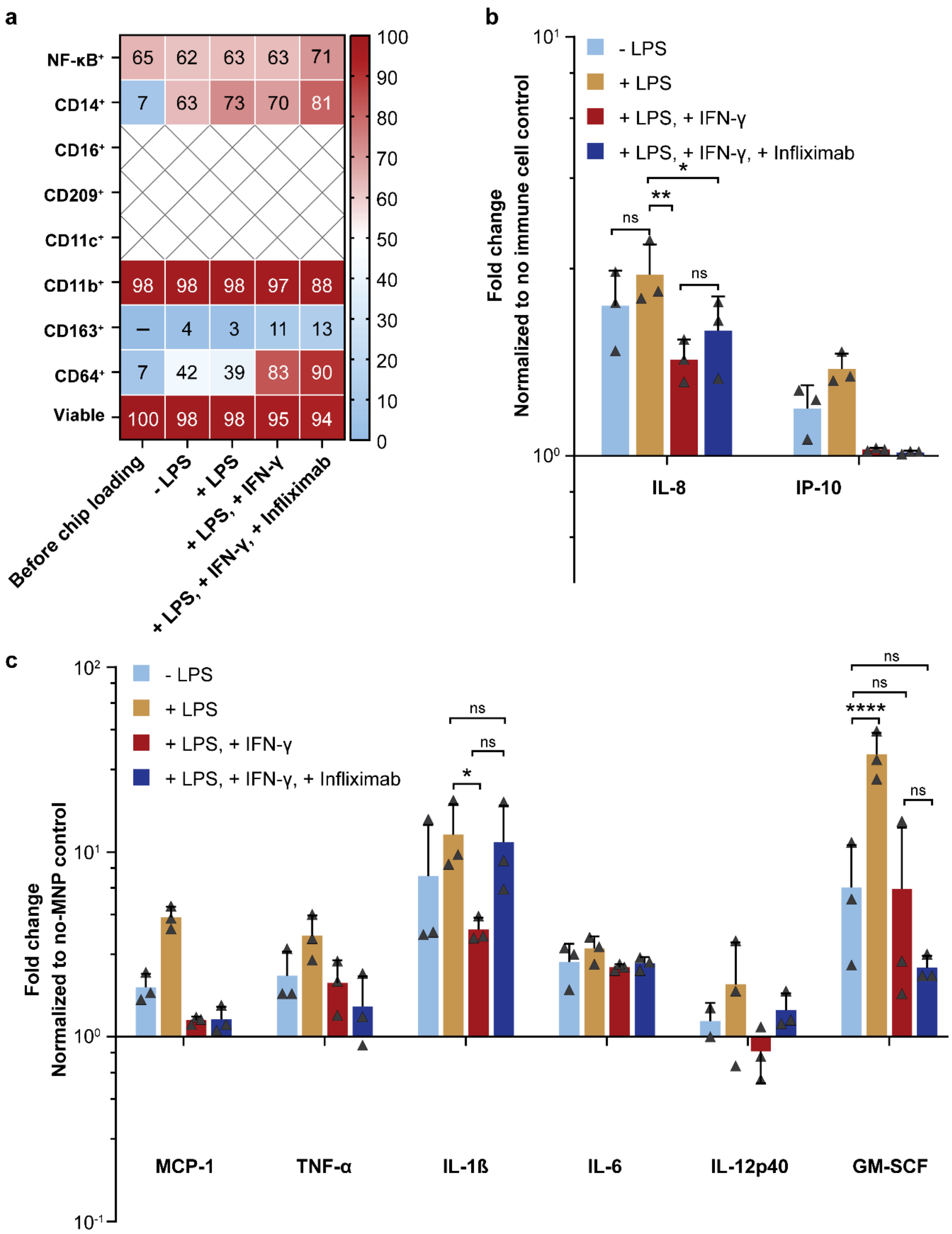


**Figure S10.** a) (i) Heat map representation of flow cytometry analysis results of the marker expression of MFs before and after 2 days in co-culture with the IEB model upon applying different stimuli. The fraction of positive cells (%) is shown in each box of the heat map. b) IEC-derived chemo/cytokine profiles in on-chip co-cultures with MFs at day 7 (n = 3). Data is represented as x-fold change with respect to the baseline of the "no-MNP" control. c) Full MF-derived cytokine profile of on-chip co-cultures of IEB models with MFs on day 7 (n = 3). Data is represented as x-fold change with respect to the baseline of the "no-MNP" control (ns: not significant, *p < 0.05, **p < 0.01, ***p < 0.001, and ****p < 0.0001).

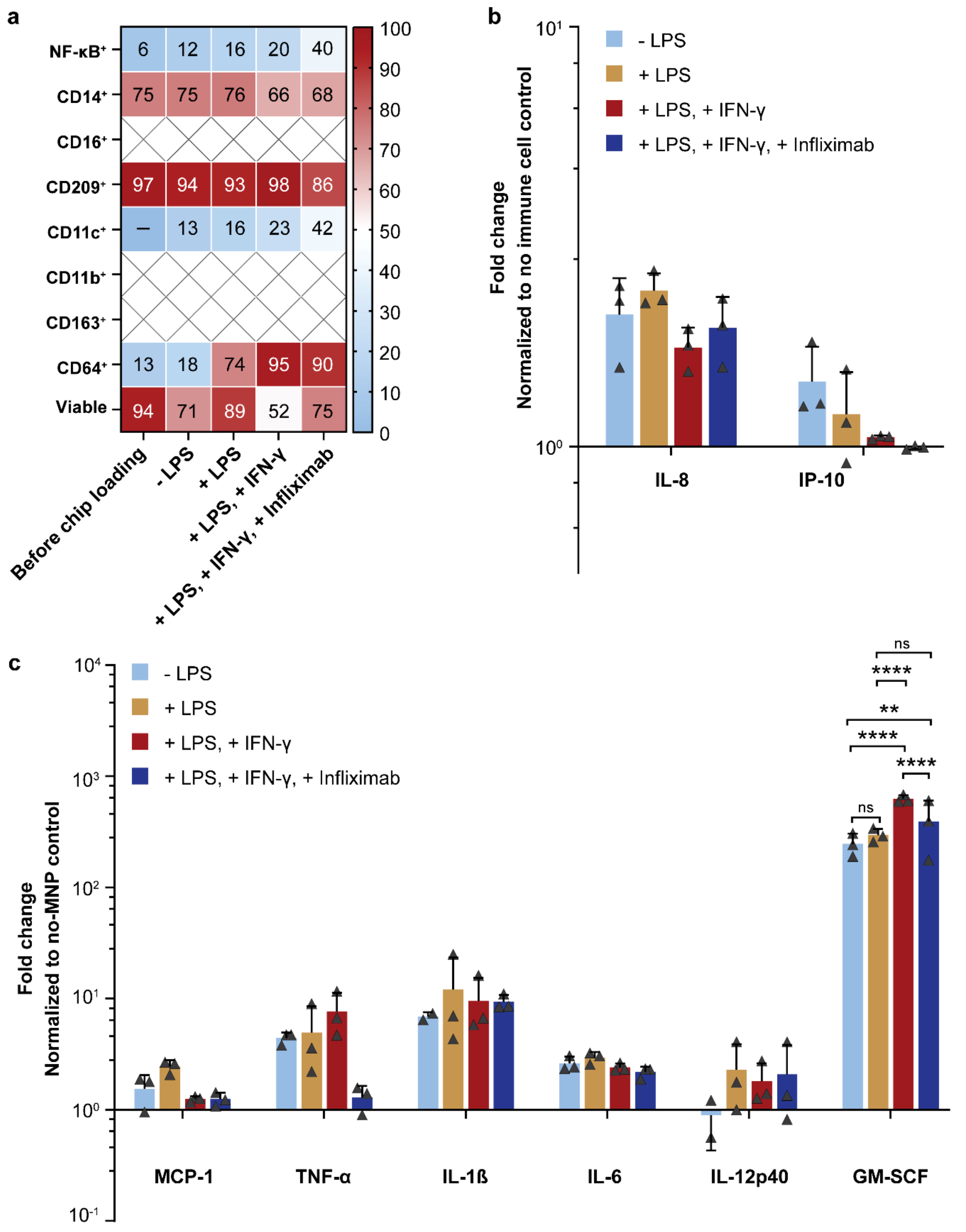


**Figure S11.** a) (i) Heat map representation of flow cytometry analysis results of the marker expression of iDCs before and after 2 days in co-cultures with the IEB model upon applying different stimuli. The fraction of positive cells (%) is shown in each box of the heat map. b) IEC-derived chemo/cytokine profiles in on-chip co-cultures with iDCs at day 7 (n = 3). Data is represented as x-fold change with respect to the baseline of "no-MNP" controls. c) Full iDC-derived cytokine profile of on-chip co-cultures of IEB models with iDCs at day 7 (n = 3). Data is represented as x-fold change with respect to the baseline of "no-MNP" controls (ns: not significant, $*p < 0.05$, $**p < 0.01$, $***p < 0.001$, and $****p < 0.0001$).

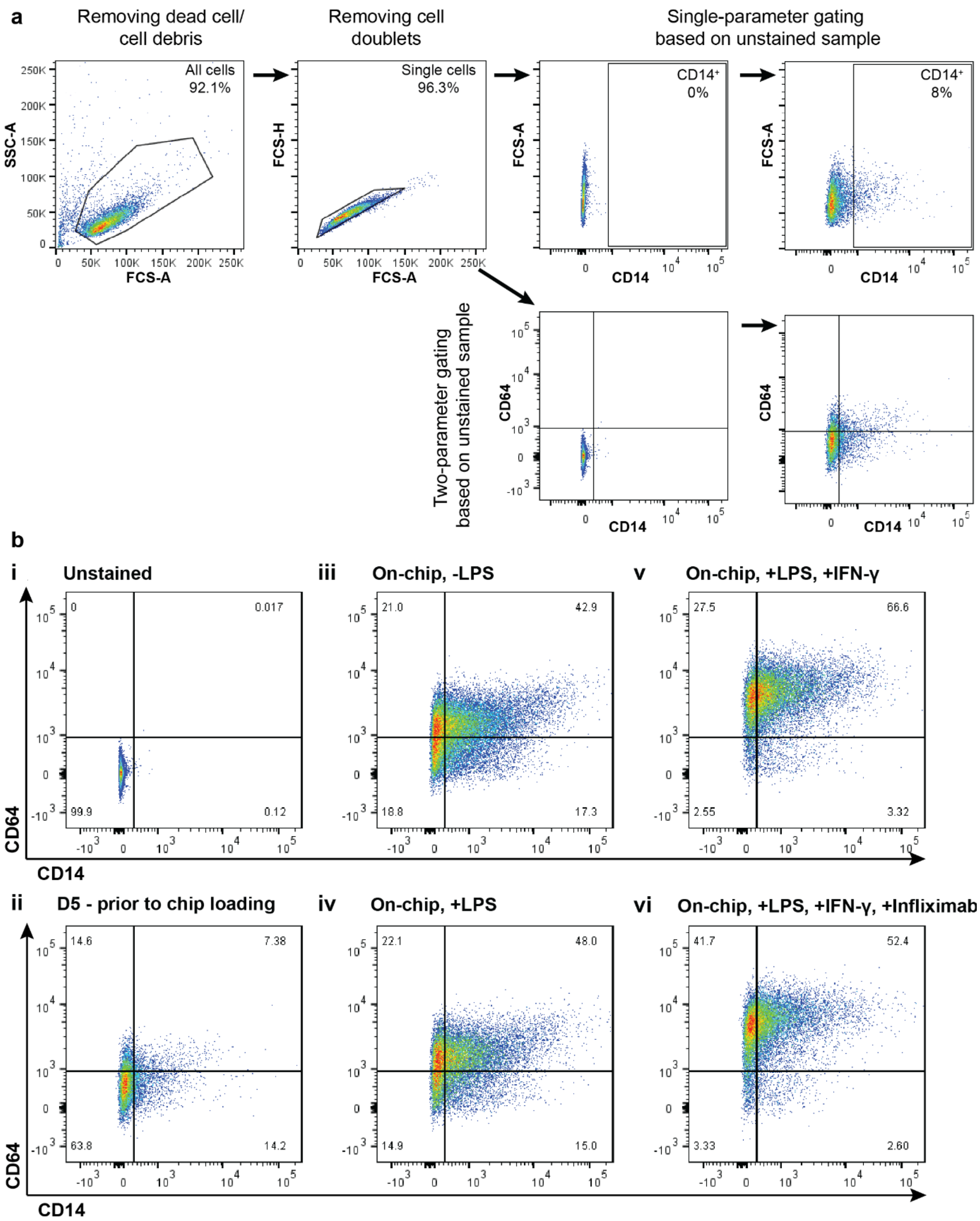


**Figure S12.** a) An example of the gating strategy used to analyze all markers. b) Flow cytometry analysis of CD14 versus CD64 expressions of MNs, shown by dot plots with pseudocolors. The gating was based on the unstained control.

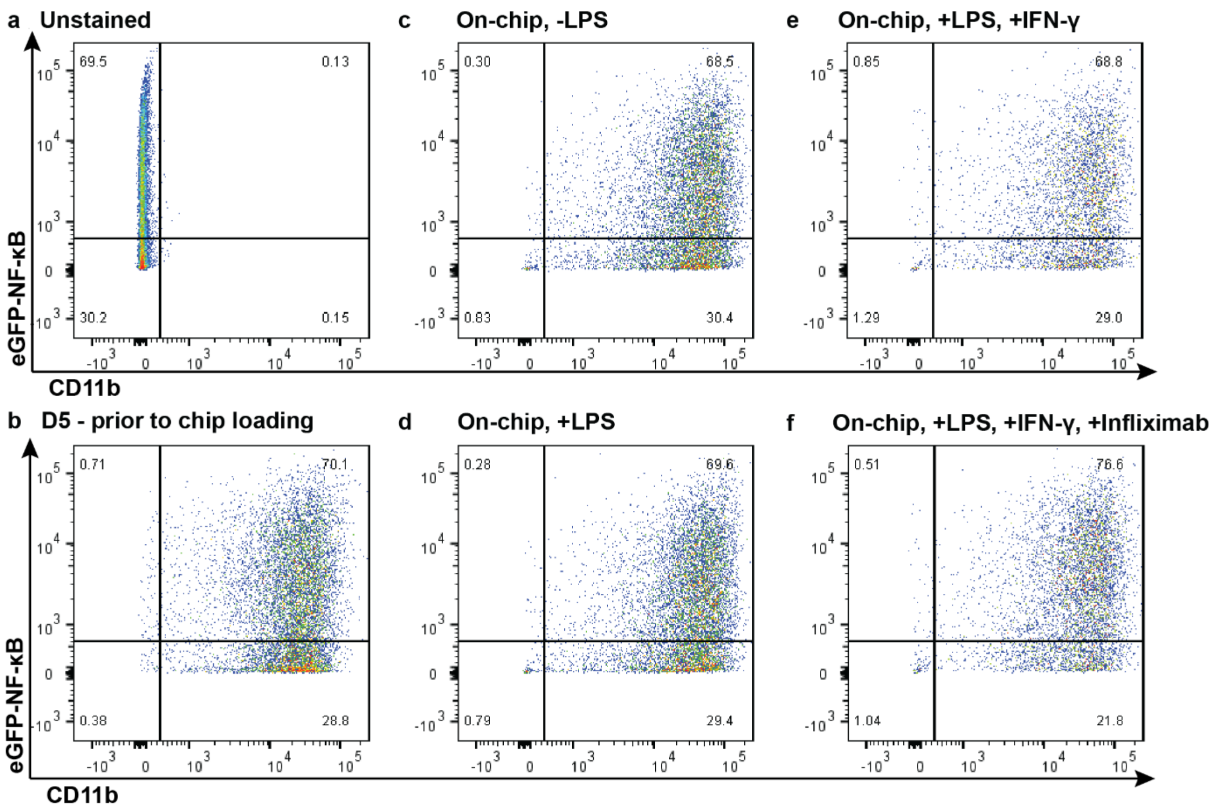


**Figure S13.** Flow cytometry analysis of CD11b versus NF-κB TF expressions of MFs, shown by pseudocolor dot plots. The gating was based on the unstained control**.**

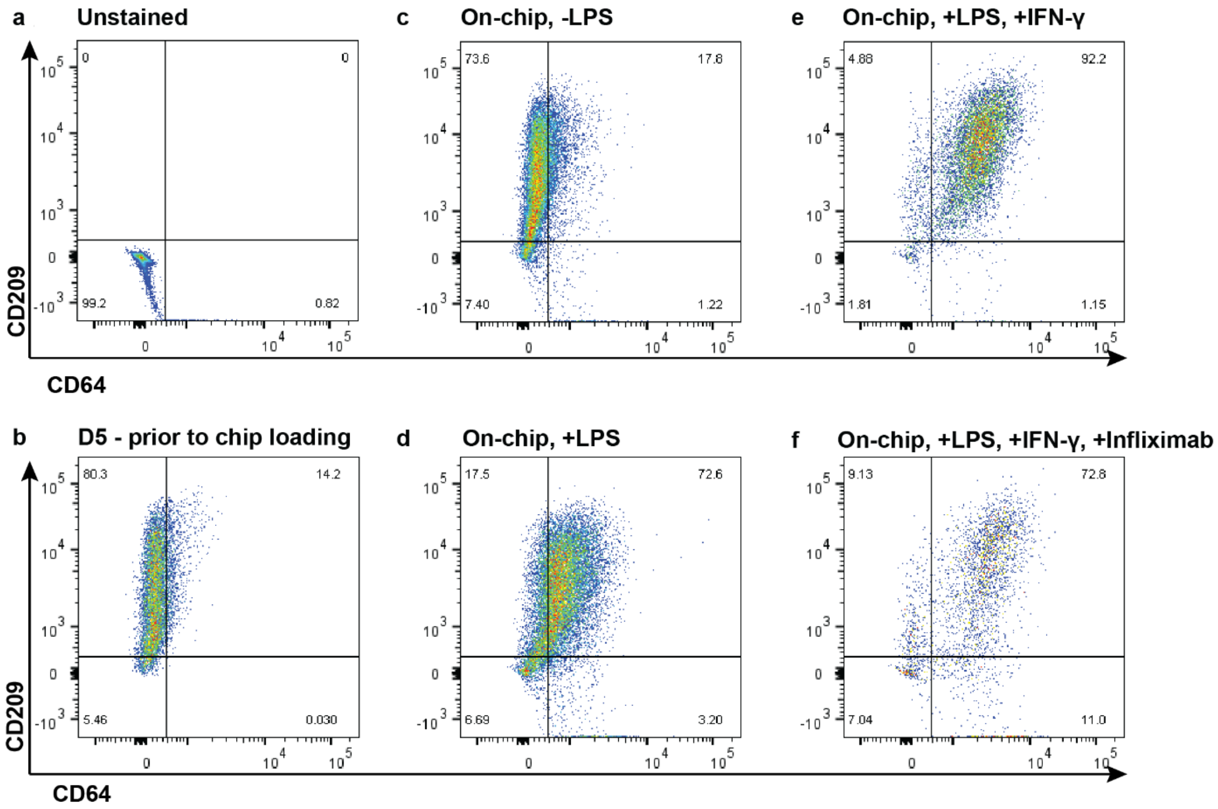


**Figure S14.** Flow cytometry analysis of CD64 versus CD209 expressions of iDCs, shown by pseudocolor dot plots. The gating was based on the unstained control.

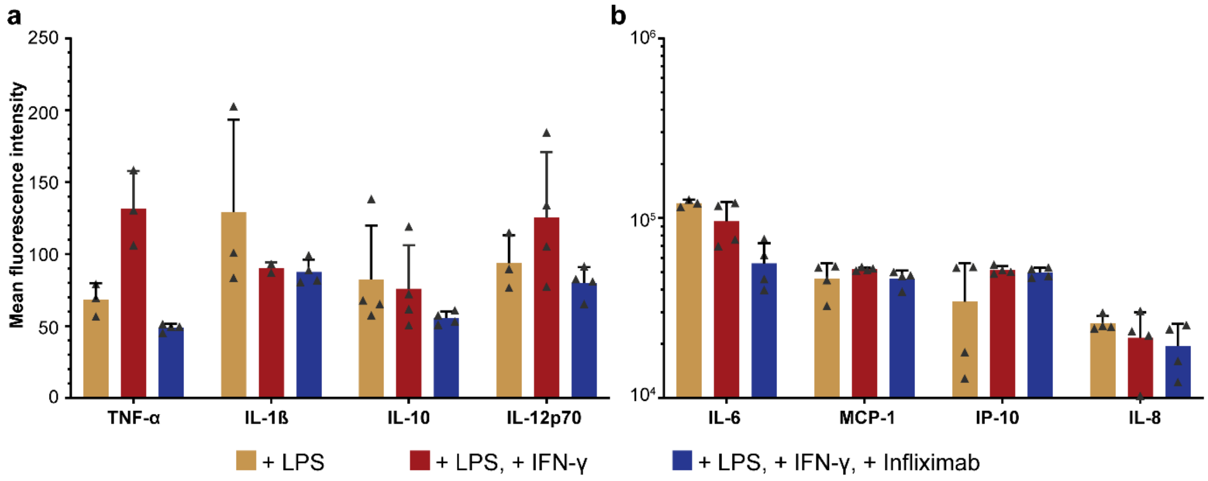


**Figure S15.** Full chemo/cytokine profiles of on-chip co-cultures of IEB models with PBMCs at day 7 ((n = 4).

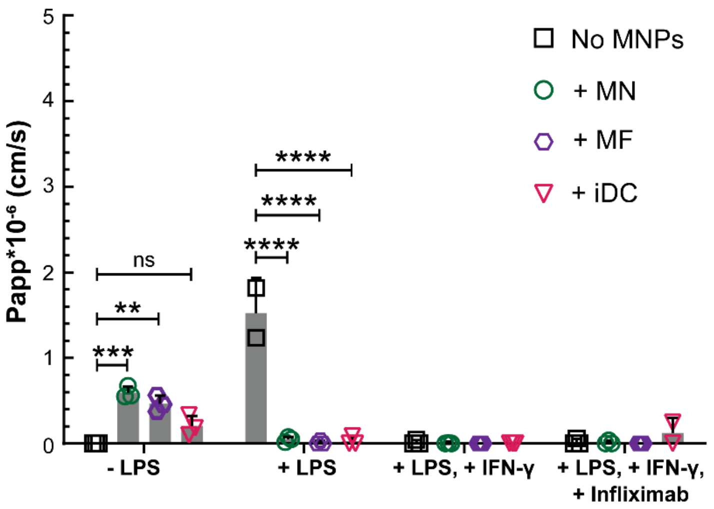


**Figure S16.** IEB model permeability for 70 kDa RITC-Dextran at day 7, shown as Papp values (n = 3). Where applicable, different types of MNPs, IFN-γ, and Infliximab were introduced to the basal compartments as shown in the timeline. Papp for only the IEB model was measured at day 5 before MNP inoculation. All other values, including Papp for the acellular CCC, were measured on day 7.

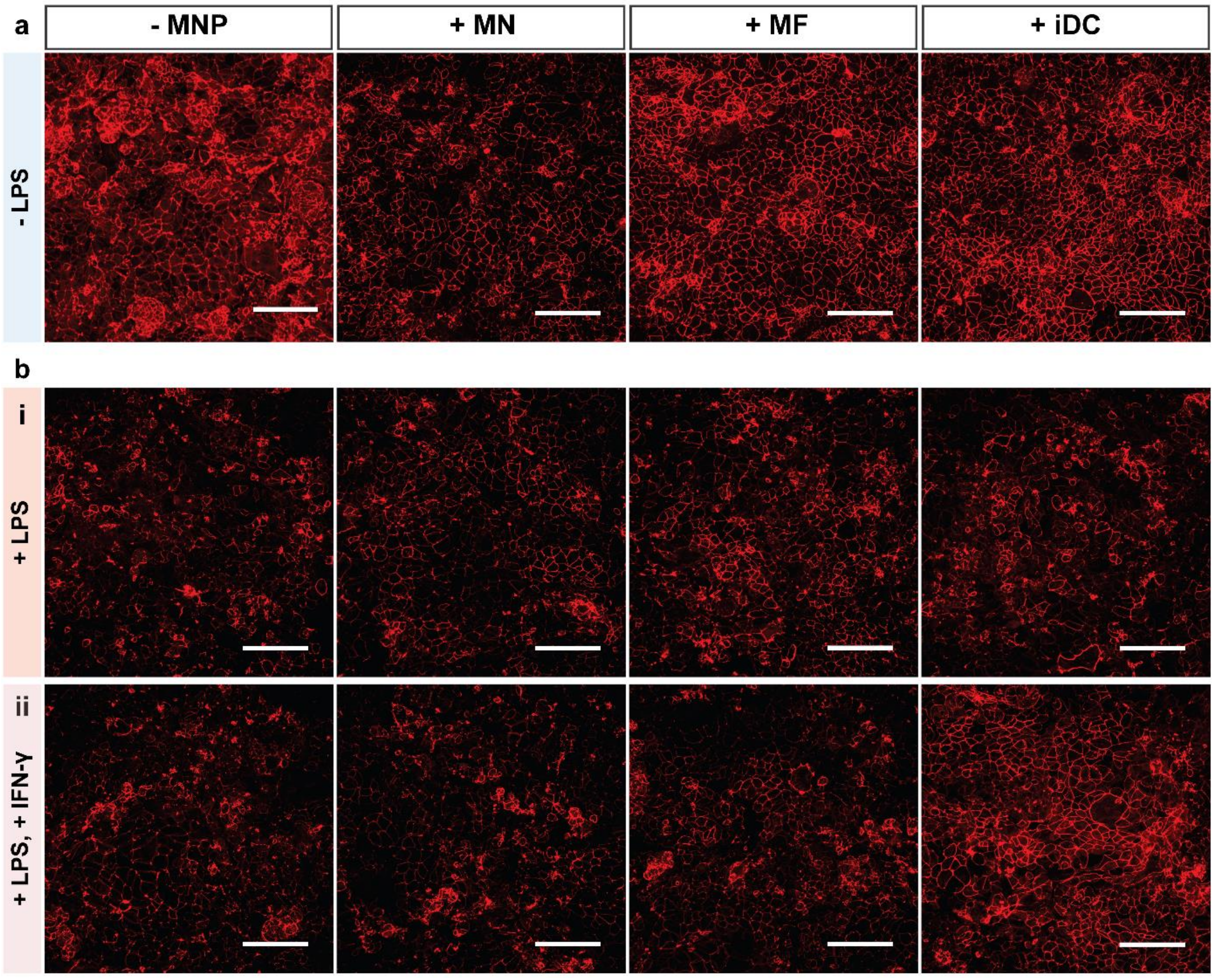


**Figure S17.** IF staining of the tight junction protein ZO-1 in on-chip IEB model-MNP co-cultures under different conditions: a) without LPS and basal stimuli, and b) (i) with apical LPS only and (ii) with apical LPS and basal IFN-γ. Scale bars: 100 μm.

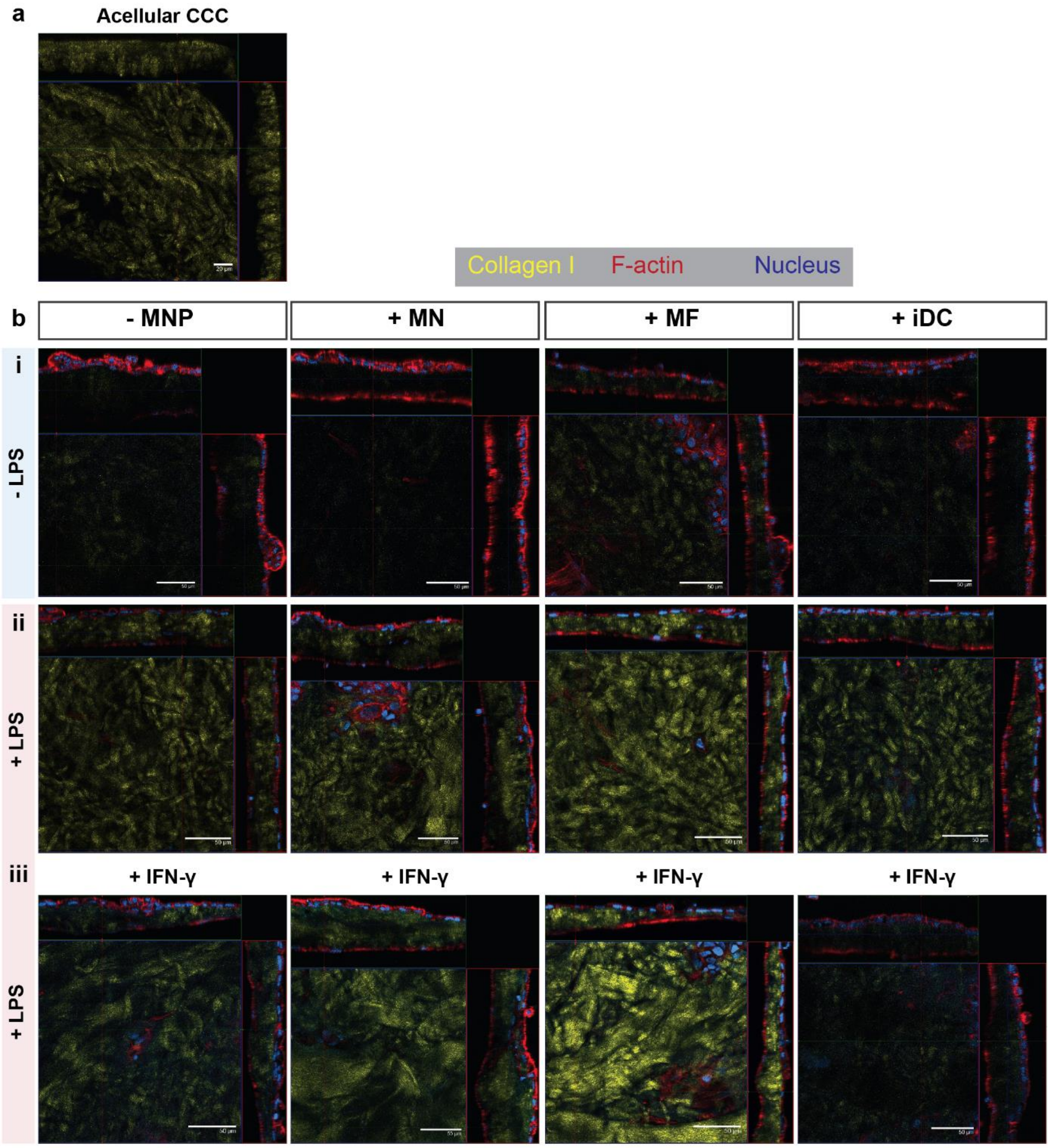


**Figure S18.** SHG microscopy and IF stainings showing the morphology and orientation of collagen I fibers with the CCC in on-chip (co-)cultures. Scale bars: 50 μm.

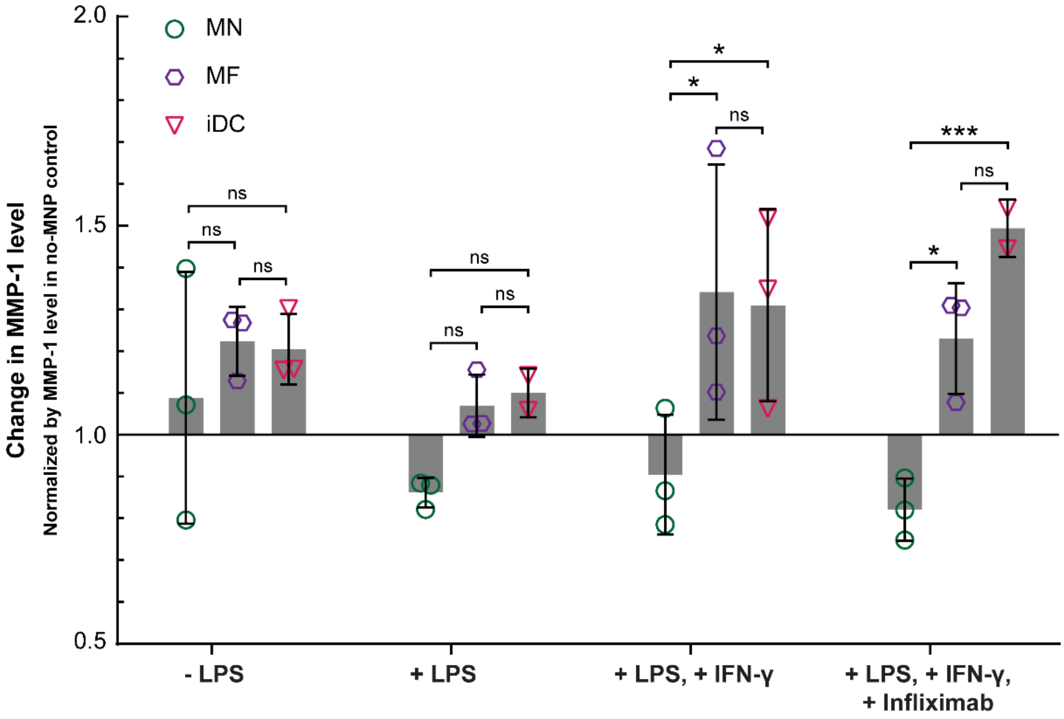


**Figure S19.** X-fold changes in MMP-1 secretion of fibroblasts in different on-chip cultures (n = 3) (ns: not significant, *p < 0.05, **p < 0.01, ***p < 0.001, and ****p < 0.0001).
